# Production of Poly(3-Hydroxybutyrate) by *Haloarcula*, *Halorubrum*, and *Natrinema* Haloarchaeal Genera Using Starch as a Carbon Source

**DOI:** 10.1155/2021/8888712

**Published:** 2021-01-26

**Authors:** Fatma Karray, Manel Ben Abdallah, Nidhal Baccar, Hatem Zaghden, Sami Sayadi

**Affiliations:** ^1^Laboratory of Environmental Bioprocesses, Centre of Biotechnology of Sfax, BP 1177, 3018 Sfax, Tunisia; ^2^Center for Sustainable Development, College of Arts and Sciences, Qatar University, Doha 2713, Qatar

## Abstract

Microbial production of bioplastics, derived from poly(3-hydroxybutyrate) (PHB), have provided a promising alternative towards plastic pollution. Compared to other extremophiles, halophilic archaea are considered as cell factories for PHB production by using renewable, inexpensive carbon sources, thus decreasing the fermentation cost. This study is aimed at screening 33 halophilic archaea isolated from three enrichment cultures from Tunisian hypersaline lake, Chott El Jerid, using starch as the sole carbon source by Nile Red/Sudan Black staining and further confirmed by PCR amplification of *phaC* and *phaE* polymerase genes. 14 isolates have been recognized as positive candidates for PHA production and detected during both seasons. The identification of these strains through 16S rRNA gene analyses showed their affiliation to *Halorubrum*, *Natrinema*, and *Haloarcula* genera. Among them, three PHB-producing strains, CEJ34-14, CEJ5-14, and CEJ48-10, related to *Halorubrum chaoviator*, *Natrinema pallidum*, and *Haloarcula tradensis* were found to be the best ones reaching values of 9.25, 7.11, and 1.42% of cell dry weight (CDW), respectively. Our findings highlighted that *Halorubrum*, *Natrinema*, and *Haloarcula* genera were promising candidates for PHB production using soluble starch as a carbon source under high salinity (250 g L^−1^ NaCl).

## 1. Introduction

Plastic is a highly useful material, and its production is growing. Million tons of nondegradable plastics end up in our natural environment every year affecting our health, wildlife, terrestrial, and marine habitats [1]. For this reason, polyhydroxyalkanoates (PHAs) are biodegradable and biocompatible polymers which have been promoted as an alternative to conventional oil-based plastics [2, 3]. PHAs are synthesized by a wide variety of bacteria and archaea from various carbon sources and served as intracellular storage compounds to survive under unbalanced conditions [4, 5]. Extremely halophilic archaea, inhabiting hypersaline environments containing high salt concentrations, are preferred for various potential applications, including the synthesis of polyhydroxyalkanoates. Poly(3-hydroxybutyrate) (PHB) was the first bioplastic found in species of Dead Sea *Halobacterium* described in 1972 [6]. Since then, microorganisms including extremely halophilic archaea belonging to the genera *Haloquadratum*, *Halorubrum*, *Halobacterium*, *Haloterrigena*, *Haloferax*, *Natronococcus*, *Natronobacterium*, *Haloarcula*, *Natrinema*, *Halogeometricum*, *Halopiger*, *Halobiforma*, and *Halococcus* have also been found to accumulate considerable amounts of PHB [7–9] using colony/cell staining methods, molecular tools targeting PHA synthase genes, and analytical techniques such as Fourier transform infrared spectroscopy, crotonic acid assays, gas chromatography, and liquid chromatography [10].

These organisms could utilize various renewable carbon sources and release the PHB easily by cell lysis in distilled water, thus lowering its high production cost [11]. Several recent investigations have shown that the productivities of PHB by archaeal strains were obtained using glucose as the sole carbon source [9, 12, 13] than with other substrates (wastes and pretreated vinasse) [14, 15]. However, few studies based on the exploration of PHB production by archaeal species using starch have been reported, despite its wide availability [16, 17]. Therefore, it was important to select strains able to use starchy substrates for PHB biosynthesis.

Chott El Jerid, the largest Salt Lake, is located in the south of Tunisia. Furthermore, this lake is the biggest one in the north of Africa (5360 km^2^) with a salt concentration above 33% NaCl [18]. It has a thalassohaline salt composition, despite its continental origin. It may be flooded in the winter and evaporates to a desert in the dry season. These climatic conditions make the Chott an ephemeral extreme environment. Our previous studies described microbial diversity in Chott El Jerid during wet and dry seasons, using culture and molecular methods. Halophilic anaerobic fermentative bacterial strains were isolated from surface sediments [19, 20]. Culture-independent techniques, targeting 16S rRNA and functional markers encoding the dissimilatory sulfite reductase beta-subunit gene (*dsrB*), methyl coenzyme M reductase (*mcrA*), showed abundant and diverse prokaryotic communities, sulfate-reducing bacteria (SRB), and methanogens, respectively [20, 21]. Additionally, the isolation of halophilic aerobic bacterial and archaeal strains producing extracellular hydrolases have been achieved in Chott El Jerid [22]. Because Archaea outnumbered Bacteria in the studied samples, our purpose, here, is to expand the possibility of PHA synthesis by Archaea from Chott El Jerid. The objectives of this research were (1) enrichment, isolation, and screening PHA-accumulating halophilic archaea in water or water/sediment mix samples collected from Chott El Jerid in both seasons using phenotypic and molecular methods; (2) identification and characterization of PHA-producing isolates, utilizing starch as the carbon source; and (3) identification and quantification of the polymer produced.

## 2. Materials and Methods

### 2.1. Sample Collection

The samples (S1-10) and (S6M-14; S6W-14) were collected in dry (October 2010) and wet seasons (January 2014) from a continental ephemeral lake, Chott El Jerid, respectively. Hypersaline water or water/sediment mix samples were collected at different locations, approximately 0-10 cm from the surface. The samples were collected in sterile bottles, brought to the laboratory within three hours, and kept aseptically at 4°C until analyses. The environmental parameters of sampling sites are listed in Table 1 as previously reported [20].

### 2.2. Enrichment and Isolation of Polyhydroxyalkanoic Acids (PHA) Producing Archaea

Samples were enriched by culturing in PHA-accumulating medium (per liter NaCl, 250 g; MgCl_2_.6 H_2_O, 10 g; MgSO_4_.7 H_2_O, 15 g; KCl, 4 g; CaCl_2_, 2 H_2_O, 1 g; NaHCO_3_, 0.5 g; yeast extract, 1 g) supplemented with 1% soluble starch at 37°C for 7 days at 180 rpm.

In order to isolate PHA-producing haloarchaea, samples were serially diluted and 100 *μ*L of each dilution were plated onto agar medium (20 g L^−1^) as described above. Following incubation, 33 colonies from the plates were picked as a result of their pigmentation and/or morphology and were transferred onto fresh plates several times until pure culture was obtained. Colonies were differentiated by colour, shape, and edge appearance. Morphological features of cells were examined using oil immersion at 100x objective (Optika, B-500 ERGO Model, Italy).

### 2.3. Extraction of Genomic DNA

When the growth of archaeal cultures reached the exponential phase, the extraction of genomic DNA was performed using the GF-1 Nucleic Acid Extraction Kit (Vivantis Technologies Sdn Bhd, Selangor DE, Malaysia) according to the manufacturer's instructions.

### 2.4. Identification of Archaeal Strains

PCR amplification was achieved using the primer set 21F (5′-TTCCGGTTGATCCYGCCGGA-3′) [23] and 1492R (5′-GGTTACCTTGTTACGACTT-3′) [24]. The PCR reaction was realized in a 50 *μ*L mixture containing 1.25 U of Taq polymerase (Fermentas), 1x PCR buffer, 200 *μ*M of dNTP, 0.2 *μ*M of each primer, and 50 ng of genomic DNA. Thirty cycles (1 min 94°C; 1 min 55°C; 2 min 72°C) were carried out using a thermocycler (Applied Biosystems, USA). The amplified PCR products of size 1500 bp were analyzed by electrophoresis in 1% agarose gels and photographed with a Gel Doc XR Imaging system (Bio-Rad). Then, the restriction analysis was performed by digesting 10 *μ*L of PCR products with 10 U of restriction enzymes *Alu*I and *Mbo*I while *Hae*III (8 U *μ*L^−1^) and the appropriate restriction buffer in a final volume of 20 *μ*L for 3 hours at 37°C. These enzymes (Life Technologies), frequently used in restriction analysis, gave the most significant differences between species. 16S rRNA fragments, acquired after enzymatic digestion, were separated on 3% agarose gels for 4 h at 50 V and photographed with a Gel Doc system. The original PCR products of positive isolates selected by comparing enzymatic restriction patterns and described as PHA-producing isolates in the following section were purified with PureLink® Quick Gel Extraction and PCR Purification Combo Kit (Cat. No. K220001, Invitrogen, Carlsbad, USA) following the manufacturer's instructions prior to cloning. The purified PCR products were ligated into *pGEM-T easy* (Promega Corporation, Madison, WI) system as recommended by the manufacturer. The ligation mixture was transformed into DH5*α* competent cells. Recombinant plasmids were verified by *Eco*RI digestion and chosen for sequencing. Sequencing and phylogenetic analysis were performed as previously reported [25].

### 2.5. Screening of Potential Halophilic PHA Producers

All isolates were subjected to PHA screening by Sudan Black B (SBB) and confirmed by Nile Red (NR) staining (Sigma), a more specific stain. Staining of colonies was done using 0.3% alcoholic solution of Sudan Black B. PHA-producing colonies appeared black [26]. Nile Red stain (25% (w/v) stock solution in dimethylsulfoxide (DMSO)) was directly inoculated in duplicate (0.5 *μ*g mL^−1^ (w/v)) in agar medium containing 1% (w/v) starch and growth cells occurred in the presence of the dye. The strain *Escherichia coli* was used as a negative control. *Natrinema altunense* strain CEJGTEA101 [KY129977] was identified as a PHA-producing isolate in our previous work [9] and was used as a positive control in this study. After 15 days of incubation at 37°C, the isolates which revealed orange fluorescence after exposure of plates to UV light were selected as PHA accumulators [27]. Staining of promising cells with Nile Red was done using a fluorescence microscope (Olympus BX51) [28].

In parallel, all isolates were screened for their genetic potential for polyhydroxyalkanoate production. The genes responsible for PHA production in haloarchaea were clustered in the class III synthases which were constituted of two subunits (*PhaE* and *PhaC*). PCR technique was used for screening archaeal polyhydroxyalkanoate producers using two pairs of codehops (codehopEF/codehopER) and (codehopCF/codehopCR), according to the highly conserved regions in *PhaE* and *PhaC*, respectively [29, 30]. The primers codehopEF (5′-CGACCGAGTTCCGCGAYATHTGGYT-3′) and codehopER (5′-GCGTGCTGGCGGCKYTCNAVYTC-3′) were used to amplify the *PhaE* polymerase gene. The amplification of the *PhaC* polymerase gene was performed using the primers codehopCF (5′-ACCGACGTCGTCTACAAGGARAAYAARYT-3′) and codehopCR (5′-GGTCGCGGACGACGTCNACRCARTT-3′) [30]. The PCR condition was as follows: after initial denaturation (94°C for 5 min), 30 cycles of 94°C for 30 s, 55°C for 45 s, and 72°C for 45 s were performed, followed by a final extension (10 min, 72°C). PCR amplification was run on a thermocycler (Applied Biosystems) using 1.25 U of Taq DNA polymerase (Fermentas), 1x PCR buffer, 0.2 *μ*M of each primer, 200 *μ*M of DNTP, and 50 ng DNA template. The PCR products were subjected to electrophoresis using 2% agarose gels.

### 2.6. Growth Kinetics of Potential PHA Producers

The strains were cultivated in 50 mL of PHA-producing medium amended with 10 g L^−1^ starch. The cultures in Erlenmeyer flasks were incubated in duplicate at 37°C, 180 rpm. Absorbance at 600 nm was determined using a UV-visible spectrophotometer (Shimadzu UV-1800, Japan) each 24 h for each PHA-producing strain.

### 2.7. Determination of Cell Dry Weight (CDW)

Cultures grown to late logarithmic phase were centrifuged at 6000 rpm for 30 min; then, cells were washed twice with distilled water. The supernatant was discarded leaving the pellet, which was lyophilized and weighed.

### 2.8. Determination of PHB Content in Potential Halophilic PHA Producers by Gas Chromatography (GC)

After methanolysis, the cellular content of the polymer and its composition were assayed by gas chromatography (GC) using an Agilent Technologies 7890A chromatograph, equipped with a capillary column (30 m × 0.25 mm × 0.25 *μ*m) and a flame ionization detector (FID) as previously reported [31]. Samples were analyzed in duplicate. The standard PHB (Sigma-Aldrich, USA) was used for calibration. The PHB content in the cells was determined as (mass of PHB/cell dry mass) × 100%. The peak at 4.4 min represents the 3-hydroxybutyrate methylester.

## 3. Results

### 3.1. Screening of PHA-Producing Isolates by Staining Procedures

A large number of orange, red, and pink colonies were picked and purified by repeating subculturing. A total of 33 extremely halophilic strains were isolated from three tested enrichment cultures and were screened for PHA accumulation (Table 2). 11, 20, and 2 isolates were obtained from the water/sediment mix of the sample S1-10, water/sediment mix of the sample S6M-14, and hypersaline water of the sample S6W-14, respectively. 14 positive isolates were selected after staining by Sudan Black B (Figure S1 (a)). Additionally, they showed high fluorescence intensity with Nile Red when exposed to ultraviolet (Figure S1 (b)). The positive control strain exhibited orange fluorescence under UV light.

### 3.2. Screening of PHA Synthase Genes by Degenerate Polymerase Chain Reaction

As a result, the screening of 33 strains showed the detection of 14 strains as PHA producers. The same strains, which showed positive results with phenotypic methods (Sudan Black and Nile Red), gave bands of approximately 230 bp (*phaE*) and 280 bp (*phaC*) (Table 2; Figure S2).

### 3.3. Morphological Characterization of Potential PHA Producers

The cells of all isolates were rods, cocci, and pleomorph (Table 2). All selected strains as producers were round. The approximate cell dimensions were 1 to 2 *μ*m (Figure S3).

### 3.4. Phylogenetic Analysis

**Figure 1:**
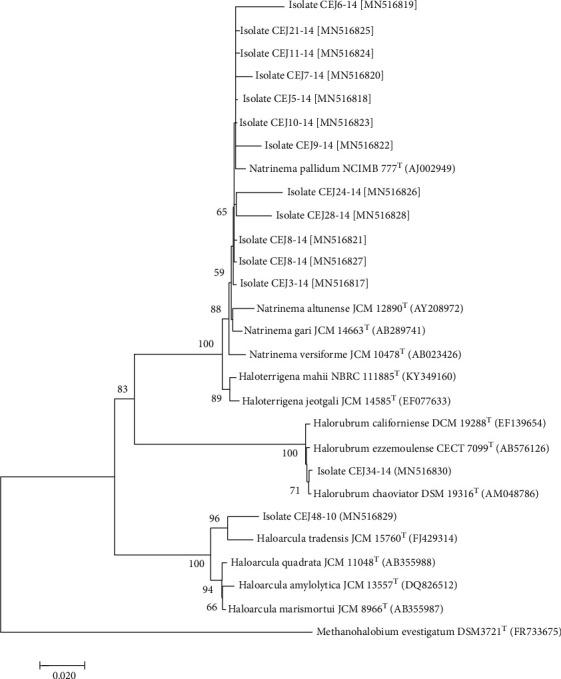
Phylogenetic tree, reconstructed by the neighbor-joining method, showing the positions of PHA-accumulating haloarchaeal isolates. Bootstrap values based on 1000 replicates are indicated. The sequence of *Methanohalobium evestigatum* was used as the outgroup.

33 haloarchaeal isolates were examined with ARDRA analysis. The comparison of three enzymes *Hae*III, *Alu*I, and *Mbo*I digestion patterns withisolates showed the occurrence of six different patterns for archaea (Table 2). Three profiles grouped polyhydroxyalkanoate-producing strains. The strains CEJ3-14, CEJ5-14, CEJ6-14, CEJ7-14, CEJ8-14, CEJ9-14, CEJ10-14, CEJ11-14, CEJ21-14, CEJ24-14, CEJ25-14, and CEJ28-14 were clustered into profile III. The strain CEJ34-14 was grouped into profile I. ARDRA pattern V represented the isolate CEJ48-10 (Figure S4). The phylogenetic analysis targeting the 16S rRNA genes of isolates indicated that the genera were *Natrinema*, *Haloarcula*, and *Halorubrum*. The isolate CEJ34-14 was related to *Halorubrum chaoviator* DSM 19316. The isolates CEJ3-14, CEJ5-14, CEJ6-14, CEJ7-14, CEJ8-14, CEJ9-14, CEJ10-14, CEJ11-14, CEJ21-14, CEJ24-14, CEJ25-14, and CEJ28-14 showed close relatedness to species of the most abundant genus *Natrinema*. Finally, the strain CEJ48-10 belonged to the genus *Haloarcula* (Figure 1).

### 3.5. Quantitative Estimation of PHB Production by Positive Haloarchaeal Isolates

**Figure 2:**
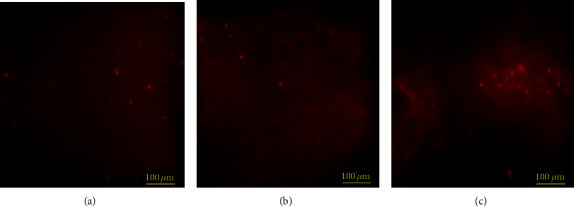
Fluorescence microscopy of archaeal cells grown on PHA accumulation medium upon 144 h of cultivation for strain CEJ34-14 (a), 168 h for strain CEJ5-14 (b), and 120 h for strain CEJ48-10 (c) following staining with Nile Red.

**Figure 3:**
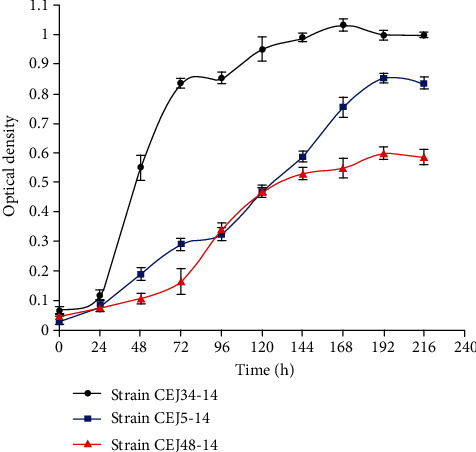
Growth curve over time of the strains CEJ34-14, CEJ5-14, and CEJ48-10. Optical density was taken every 24 h at 600 nm. Mean values from duplicate tests are shown.

**Figure 4:**
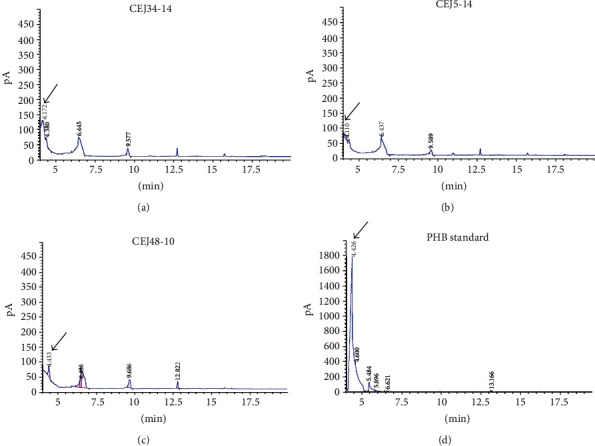
Chromatograms of PHA obtained from cultures of the isolates (a) CEJ34-14, (b) CEJ5-14, (c) CEJ48-10, and (d) PHB standard (Sigma). The peak at 4.4 min represents the 3-hydroxybutyrate methylester.

The data in Table 3 showed that the PHB content in the cells was ranged from 0.07% up to 9.25% of CDW. Among the strains studied, the isolates CEJ34-14, CEJ5-14, and CEJ48-10 affiliated with *Halorubrum chaoviator* (99.7% of similarity), *Natrinema pallidum* (99.33% of similarity), and *Haloarcula tradensis* (97.72% of similarity) were found to be the best ones, respectively. Their cells were observed under a fluorescence microscope (Figure 2). The isolate CEJ34-14 should be highlighted because it exhibited a higher PHB production and a higher growth rate after 48 h of culture. Its growth increased with a logarithmic phase of 2 days and attained a long stationary phase. However, the growth of strains CEJ5-14 and CEJ48-10 requested 3 or 4 days to reach the logarithmic phase (Figure 3). Remarkably, the strains CEJ8-14, CEJ9-14, and CEJ28-14 exhibited a higher biomass production (1150–3620 mg L^−1^) but a lower PHB production (Table 3). Comparing to the standard PHB, the GC spectrum of PHA obtained by the isolates CEJ34-14, CEJ5-14, and CEJ48-10 showed predominant peaks at retention time at 4.17, 4.11, and 4.43 minutes corresponding to poly(3-hydroxybutyrate), respectively (Figure 4). These strains exhibited PHB content of about 9.25, 7.11, and 1.42% of the cell dry weight, respectively. The growth curves and chromatograms of the other strains were presented in supplementary materials (Figures S5 and S6).

## 4. Discussion

The development of biodegradable plastics represents an alternative way to respond to problems associated with plastic waste. Polyhydroxyalkanoates are considered to be excellent candidates for biodegradable plastics. Extremely halophilic archaea have the ability to synthesize and accumulate PHA as inclusions in their cells [11, 32]. In this study, an effort has been taken to search the PHB-producing archaea isolated from the hypersaline lake, Chott El Jerid, using a starchy substrate. A total of 33 isolates were obtained from three enrichment cultures. PHB was detected in 14 strains grouped in three haloarchaeal genera *Haloarcula*, *Halorubrum*, and *Natrinema* and clustered within the phylum *Euryarchaeota* including *Haloarculaceae*, *Halorubraceae*, and *Natrialbaceae* families, respectively. They were screened via staining means (Sudan Black B and Nile Red) which were widely used for halophilic bacteria but also successfully used for *Halococci*, *Haloarcula*, *Haloferax*, *Halorubrum*, *Natronococcus*, *Halogeometricum*, *Halobacterium* genera, and other haloarchaeal strains [8, 13]. In parallel, it was evident that the detection of *phaC* and *phaE* genes were shown in the 14 archaeal cells as described above with staining methods, confirming the PHB biosynthesis. To our knowledge, few studies based on molecular characterization of the genes involved in PHB synthesis in the domain of Archaea have been investigated [33]. Importantly, one group of extremely halophilic archaea with great biotechnological importance was *Haloarcula* which was genetically well understood. Han et al. [12] identified two adjacent genes *phaE*_Hm_ and *phaC*_Hm_ encoding two subunits of PHA synthase (class III) and showed that these genes are required for PHB synthesis in *Haloarcula marismortui* (cultivated on 2% glucose, production of 21% PHB of CDW) [12]. Later, Han et al. [30] confirmed the detection of *phaEC* genes in 18 PHB or poly(3-hydroxybutyrate-co-hydroxyvalerate) PHBV producers, including *Haloarcula*, *Halorubrum*, *Natrinema*, and other genera by utilizing carbohydrates either glucose or fructose [30]. More recently, it was reported that these two genes were detected in the genome of *Natrinema altunense* CEJGTEA101, isolated from Chott El Jerid [9].

The present study is a continuity of our previous work [9], proposing the possibility to enlarge our knowledge about PHB secretion by a large number of haloarchaeal strains from Chott El Jerid due to their several advantages: firstly, their growth at high salinity minimized microbial contamination. Secondly, the high osmotic pressure in their cells facilitated the PHB recovery. Finally, their ability to consume a wide range of low-cost carbon sources reduced the PHB production cost. Among the tested isolates, three strains CEJ34-14, CEJ5-14, and CEJ48-10 affiliated with *Halorubrum chaoviator* (99.7% of similarity), *Natrinema pallidum* (99.33% of similarity), and *Haloarcula tradensis* (97.72% of similarity) have been considered as the best PHB producers 9.25%, 7.11%, and 1.42% of its CDW, respectively. It was important to note that these three genera were found to be PHA accumulators in other hypersaline environments [7], but only a few species were able to utilize starch to secrete large amounts of PHB. With regard to *Haloarcula* investigations, no PHB was accumulated by *Haloarcula* species using starch except *Haloarcula*. sp. IRU1 which could produce 57% PHB/CDW [17]. This species isolated from Urmia lake has been shown to produce important quantities of PHB (63% of CDW) from petrochemical wastewater as a carbon source containing multiple hydrocarbons such as linear alkylbenzenes [34]. Other *Haloarcula* species such as *Haloarcula japonica*, *Haloarcula amylolytica*, and *Haloarcula argentinensis* can accumulate PHB, and their yields obtained from glucose were 0.5, 4.4, and 6.5% (of CDW), respectively [30, 35]. Although production of PHB has been reported from starch, glucose, and waste materials, members of *Haloarcula* were observed for the first time in southern Tunisian salt lakes as PHB producers [15]. Currently, there is no evidence of PHB accumulation by the *Halorubrum* species when starch was used as a carbon source [11]. As previously stated, two species affiliated with *Halorubrum* which produced PHB or PHBV (2.1-12.7% of CDW) were able to use glucose as the sole carbon source [30]. On the other hand, the majority of members of *Natrinema* were found to be PHBV producers when cultivated on a medium with glucose [30] or starch [36] as the carbon sources.

In our previous work [9], 20 extremely halophilic archaea belonging to the genera *Halorubrum* (17 strains), *Natrinema* (2 strains), and *Haloterrigena* (1 strain) and isolated from the sample S1-10 collected from Chott El Jerid in the dry season were screened for PHA production in the same PHA-accumulating medium used in this study. Among them, only two strains belonging to *Natrinema* and *Haloterrigena* genera have been shown to accumulate 7% of PHB and 3.6% of poly(3-hydroxyvalerate) (PHV), respectively, in a medium supplemented with 2% glucose. In this current study, enrichment cultures with the same sample (S1-10) and supplemented with starch showed the selection of only one PHB producer related to the genus *Haloarcula*. However, enrichment cultures with samples collected in the wet season and supplemented with the same carbon source revealed the presence of one species *Halorubrum* and a high number of *Natrinema* species (12 strains) with PHB-producing ability from the samples S6W-14 and S6M-14, respectively. These findings displayed that isolation method, carbon source, season, and sampling location could influence the selection of PHB-producing archaeal species.

## 5. Conclusions

On the basis of data obtained, different archaeal isolates were obtained in pure cultures from the Tunisian desert and were able to consume starch as the sole carbon source for the PHB biosynthesis. However, the yields of PHB production in flasks batch cultures within the cells were low in comparison with other closest relatives. Therefore, future studies on fermentations optimization in batch, fed-batch, and continuous cultures using starchy and other low-cost feedstock's as well as the metabolic engineering strategies to improve both the quality and PHB productivity will be investigated.

## Figures and Tables

**Table 1:** Sampling locations, conditions, and distribution of halophilic isolates.

Sample type	Sample	Site	Geographical location	pH	Salinity (%)	Temperature (°C)	Total number of isolates
Water and sediments	S1-10	Site 1	33° 54′ 42.21^″^ N 8° 31′ 7.98^″^ E	6.61	34.6	23	11
Water and sediments	S6M-14	Site 2	33° 54′ 44.15^″^ N 8° 31′ 9.01^″^ E	7.61	27.6	19	20
Hypersaline water	S6W-14	Site 2	33° 54′ 44.15^″^ N 8° 31′9.01^″^ E	7.61	27.6	19	2

**Table 2:** Screening of PHA-producing archaeal strains using phenotypic and genotypic methods.

Sample type	Name of isolates	Morphology	Colony staining method		CODEHOP PCR		ARDRA
			SBB	NR	*PhaE*	*PhaC*	Profiles
Water and sediments (2014)	CEJ1-14	Cocci	-	-	-	-	I
	CEJ2-14	Cocci	-	-	-	-	II
	CEJ3-14	Cocci	+	+	+	+	III
	CEJ4-14	Cocci	-	-	-	-	I
	CEJ5-14	Cocci	+	+	+	+	III
	CEJ6-14	Cocci	+	+	+	+	III
	CEJ7-14	Cocci	+	+	+	+	III
	CEJ8-14	Cocci	+	+	+	+	III
	CEJ9-14	Cocci	+	+	+	+	III
	CEJ10-14	Cocci	+	+	+	+	III
	CEJ11-14	Cocci	+	+	+	+	III
	CEJ17-14	Pleomorph	-	-	-	-	I
	CEJ18-14	Pleomorph	-	-	-	-	I
	CEJ21-14	Cocci	+	+	+	+	III
	CEJ24-14	Cocci	+	+	+	+	III
	CEJ25-14	Cocci	+	+	+	+	III
	CEJ26-14	Cocci	-	-	-	-	I
	CEJ28-14	Cocci	+	+	+	+	III
	CEJ29-14	Short rod	-	-	-	-	I
	CEJ32-14	Cocci	-	-	-	-	II

Water (2014)	CEJ33-14	Cocci	-	-	-	-	I
	CEJ34-14	Cocci	+	+	+	+	I

Water and sediments (2010)	CEJ35-10	Cocci	-	-	-	-	IV
	CEJ36-10	Cocci	-	-	-	-	IV
	CEJ37-10	Cocci	-	-	-	-	IV
	CEJ38-10	Cocci	-	-	-	-	IV
	CEJ41-10	Cocci	-	-	-	-	VI
	CEJ42-10	Cocci	-	-	-	-	VI
	CEJ43-10	Cocci	-	-	-	-	IV
	CEJ45-10	Cocci	-	-	-	-	IV
	CEJ46-10	Cocci	-	-	-	-	IV
	CEJ47-10	Cocci	-	-	-	-	I
	CEJ48-10	Cocci	+	+	+	+	V

Designation of isolates by letters indicating first the origin of strains from Chott El Jerid (CEJ) followed by the number of the isolates, then a number indicating the year of sampling. The ARDRA pattern is indicated by Roman numeral. +, detectable; -, not detectable; SBB: Sudan Black B; NR: Nile Red.

**Table 3:** PHB production values by isolates and its closest relatives.

Strain^a^	Carbon source	Time^b^(h)	CDW(mg L^−1^)	PHA content^c^ (%)	Type of PHA	References
CEJ3-14	Starch	120	380	0.38	PHB	This study
CEJ5-14		168	560	7.11		
CEJ6-14		96	110	0.09		
CEJ7-14		96	560	0.69		
CEJ8-14		96	1150	0.07		
CEJ9-14		144	2500	0.1		
CEJ10-14		72	130	0.07		
CEJ11-14		96	480	0.91		
CEJ21-14		96	100	0.79		
CEJ24-14		96	300	0.89		
CEJ25-14		120	640	0.71		
CEJ28-14		96	3620	0.21		
CEJ34-14		144	220	9.25		
CEJ48-10		120	550	1.42		

*Haloarcula marismortui* ATCC 43049	Glucose	192	n.d.	21	PHB	[12]
*Haloarcula amylolytica* 26-3		96	2500	4.4	PHBV	[30]
*Haloarcula argentinensis* CGMCC 1.7094			3300	6.5	PHBV	

*Halorubrum litoreum* 12-2	Glucose	96	2500	2.1	PHB	[30]
*Halorubrum trapanicum* CGMCC 1.2201			1900	12.7	PHBV	
*Natrinema altunense* CGMCC 1.3731		96	5800	9.1	PHBV	[30]
*Natrinema pallidum* JCM 8980			3500	22.9	PHBV	
*Natrinema pellirubrum* JCM 10476			2200	11.5	PHB	
*Natrinema* sp. XA3-1			1600	5.4	PHBV	
*Natrinema ajinwuensis* RMG10		72	n.d.	61	PHBV	[37]

*Natrinema pallidum* 1KYS1	Starch	n.d.	75	53.14	PHBV	[36]
*Natrinema altunense* CEJGTEA101	Starch	120	80	2.7	PHA	[9]

CDW: cell dry weight; PHA: polyhydroxyalkanoate; PHB: poly(3-hydroxybutyrate); PHBV: poly(3-hydroxybutyrate-co-hydroxyvalerate); n.d.: not determined. ^a^Incubated at 37°C in PHA accumulation medium supplemented with starch or glucose. ^b^Cells were harvested at the early stationary phase for each strain. ^c^PHA content in dried cells was determined using gas chromatography.

## Data Availability

The sequences have been submitted to the GenBank database under accession numbers: MN516817 to MN516830.

## Supplementary Materials

Supplementary 1 Figure S1: Cells accumulating polyhydroxyalkanoic acid staining with Sudan Black B (a) and with Nile Red (b) on agar plates. The strains *Escherichia coli* and *Natrinema altunense* strain CEJGTEA101 were used as negative and positive controls, respectively.

Supplementary 2 Figure S2: PCR amplification of *PhaC* (a) and *PhaE* (b) gene encoding for PHA synthase (class III) of positive producing strains. Lane M1 represents the molecular size marker (100 bp DNA ladder) and Lane M (1 Kb DNA ladder).

Supplementary 3 Figure S3: Phase-contrast micrographs showing cells of PHA-producing strains grown in PHA-accumulating medium at 25% (w/v) NaCl; bar, 10 *μ*m.

Supplementary 4 Figure S4: (A) Restriction digestion of amplified 16S rRNA of isolates cleaving with *Alu*I (a), *Mbo*I (b), and *Hae*III (c) from the sample S1-10 and (B) from the samples S6M-14 and S6W-14. Lane M represents molecular size marker 1 Kb DNA ladder.

Supplementary 5 Figure S5: Growth curve over time of the isolates. Optical density was taken every 24 h at 600 nm. Mean values from duplicate tests are shown.

Supplementary 6 Figure S6: Chromatograms of PHB obtained from cultures of the isolates (a) CEJ3-14, (b) CEJ6-14, (c) CEJ7-14, (d) CEJ8-14, (e) CEJ9-14, (f) CEJ10-14, (g) CEJ11-14, (h) CEJ21-14, (i) CEJ24-14, (j) CEJ25-14, (k) CEJ28-14, and (l) PHB standard (Sigma).
