# Supplementary material for: Production of Poly(3-Hydroxybutyrate) by Haloarcula, Halorubrum, and Natrinema Haloarchaeal Genera Using Starch as a Carbon Source
Source: Archaea. 2021 Jan 26;2021:8888712. doi: 10.1155/2021/8888712 (PMC7860971; doi:10.1155/2021/8888712)
Supplement: Supplementary 1 — Figure S1: Cells accumulating polyhydroxyalkanoic acid staining with Sudan Black B (a) and with Nile Red (b) on agar plates. The strains Escherichia coli and Natrinema altunense strain CEJGTEA101 were used as negative and positive controls, respectively. [file 8888712.f1.docx]

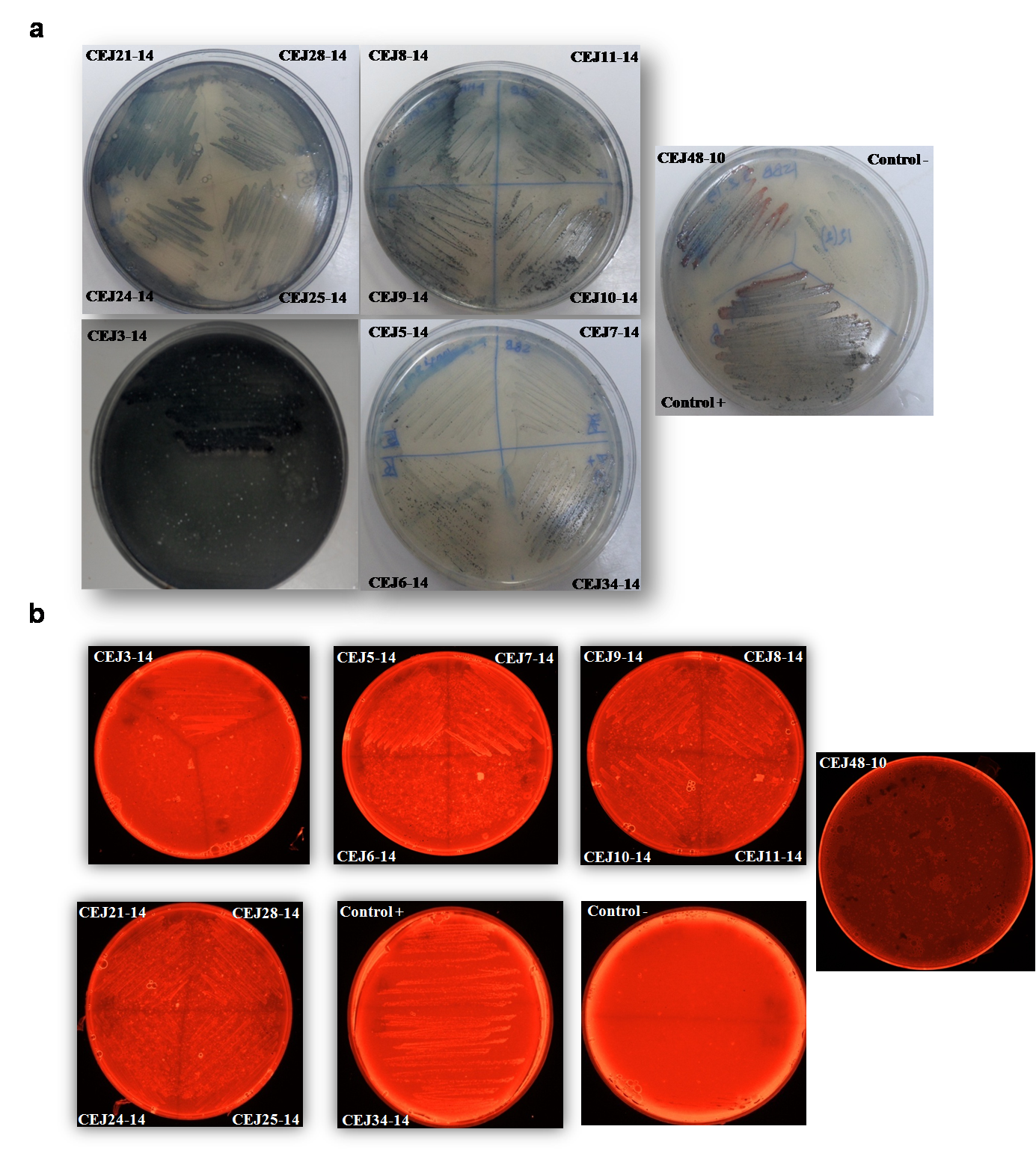


Figure S1: Cells accumulating polyhydroxyalkanoic acids staining with Sudan Black B (a) and with Nile Red (b) on agar plates. The strains *Escherichia coli* and *Natrinema altunense* strain CEJGTEA101 were used as negative and positive controls, respectively.
