# Supplementary material for: Production of Poly(3-Hydroxybutyrate) by Haloarcula, Halorubrum, and Natrinema Haloarchaeal Genera Using Starch as a Carbon Source
Source: Archaea. 2021 Jan 26;2021:8888712. doi: 10.1155/2021/8888712 (PMC7860971; doi:10.1155/2021/8888712)
Supplement: Supplementary 2 — Figure S2: PCR amplification of PhaC (a) and PhaE (b) gene encoding for PHA synthase (class III) of positive producing strains. Lane M1 represents the molecular size marker (100 bp DNA ladder) and Lane M (1 Kb DNA ladder). [file 8888712.f2.docx]

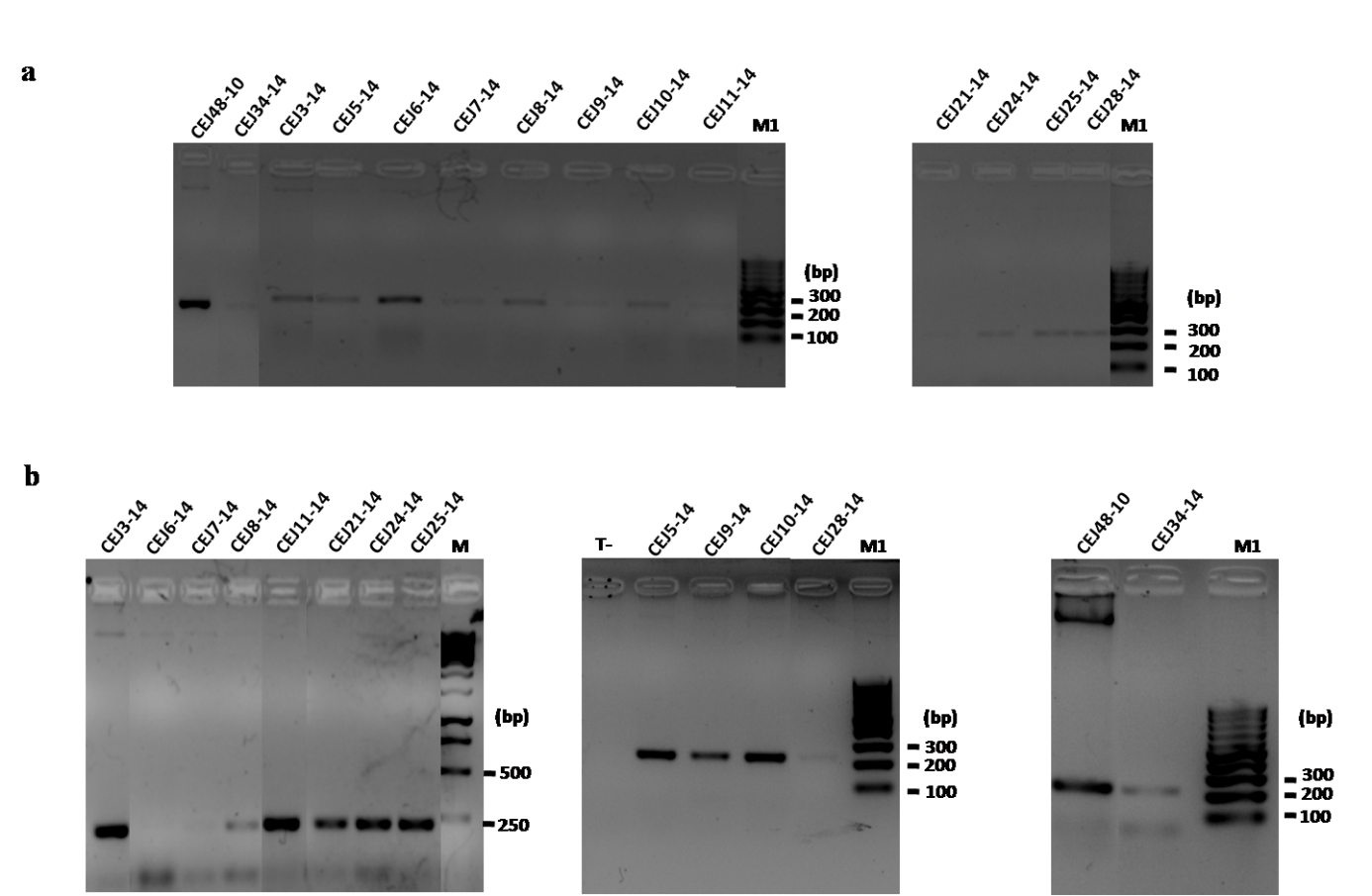


Figure S2: PCR amplification of *PhaC* (a) and *PhaE* (b) genes encoding for PHA synthase (classe III) of positive producing strains. Lane M1 represents molecular size marker (100 bp DNA ladder) and Lane M (1 Kb DNA ladder).
