# Supplementary material for: Production of Poly(3-Hydroxybutyrate) by Haloarcula, Halorubrum, and Natrinema Haloarchaeal Genera Using Starch as a Carbon Source
Source: Archaea. 2021 Jan 26;2021:8888712. doi: 10.1155/2021/8888712 (PMC7860971; doi:10.1155/2021/8888712)
Supplement: Supplementary 3 — Figure S3: Phase-contrast micrographs showing cells of PHA-producing strains grown in PHA-accumulating medium at 25% (w/v) NaCl; bar, 10 μm. [file 8888712.f3.docx]

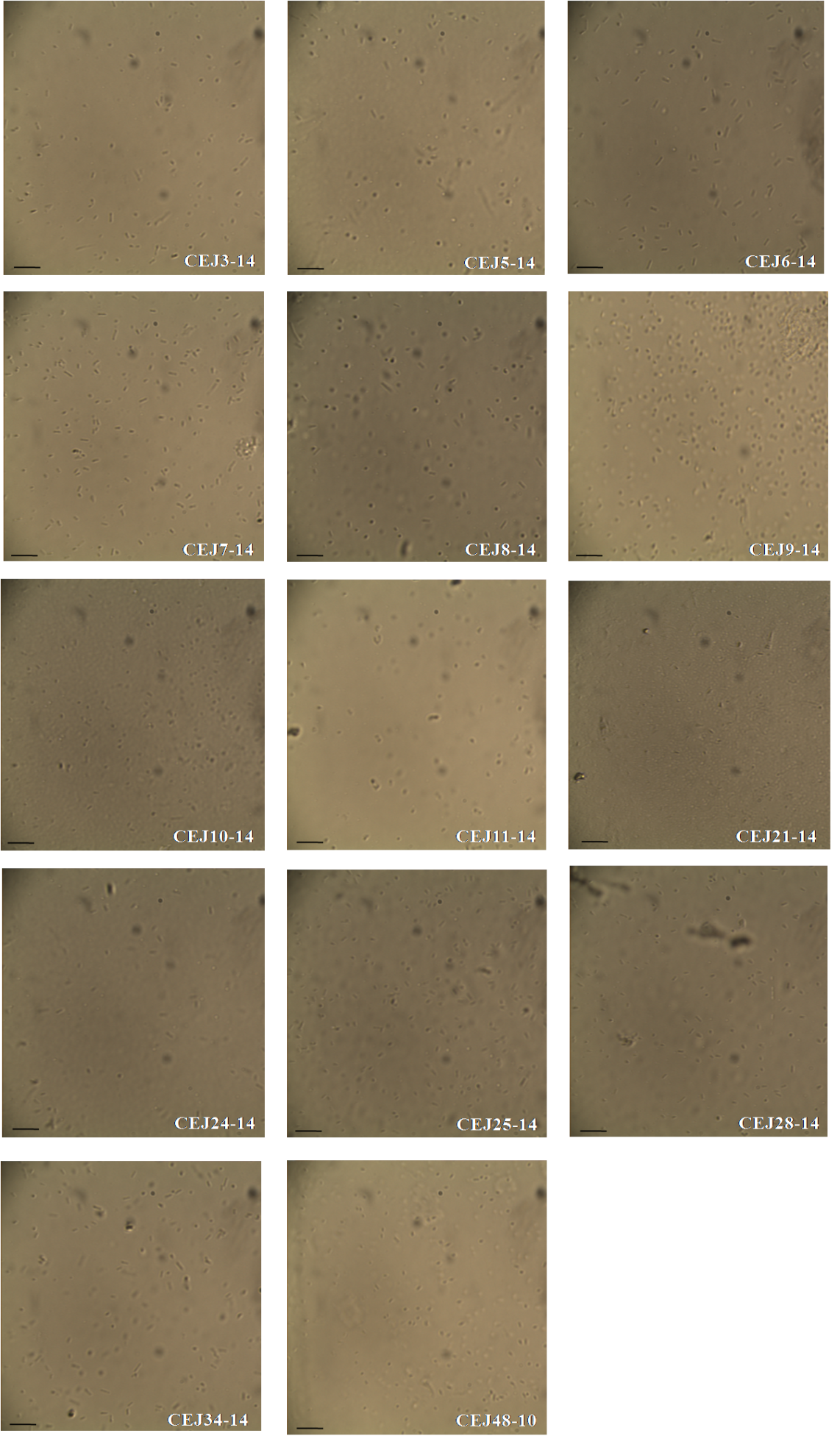


**Figure S3:** Phase-contrast micrographs showing cells of PHA-producing strains grown in PHA-accumulating medium at 25 % (w/v) NaCl; bar, 10 µm.
