# Supplementary material for: Production of Poly(3-Hydroxybutyrate) by Haloarcula, Halorubrum, and Natrinema Haloarchaeal Genera Using Starch as a Carbon Source
Source: Archaea. 2021 Jan 26;2021:8888712. doi: 10.1155/2021/8888712 (PMC7860971; doi:10.1155/2021/8888712)
Supplement: Supplementary 4 — Figure S4: (A) Restriction digestion of amplified 16S rRNA of isolates cleaving with AluI (a), MboI (b), and HaeIII (c) from the sample S1-10 and (B) from the samples S6M-14 and S6W-14. Lane M represents molecular size marker 1 Kb DNA ladder. [file 8888712.f4.docx]

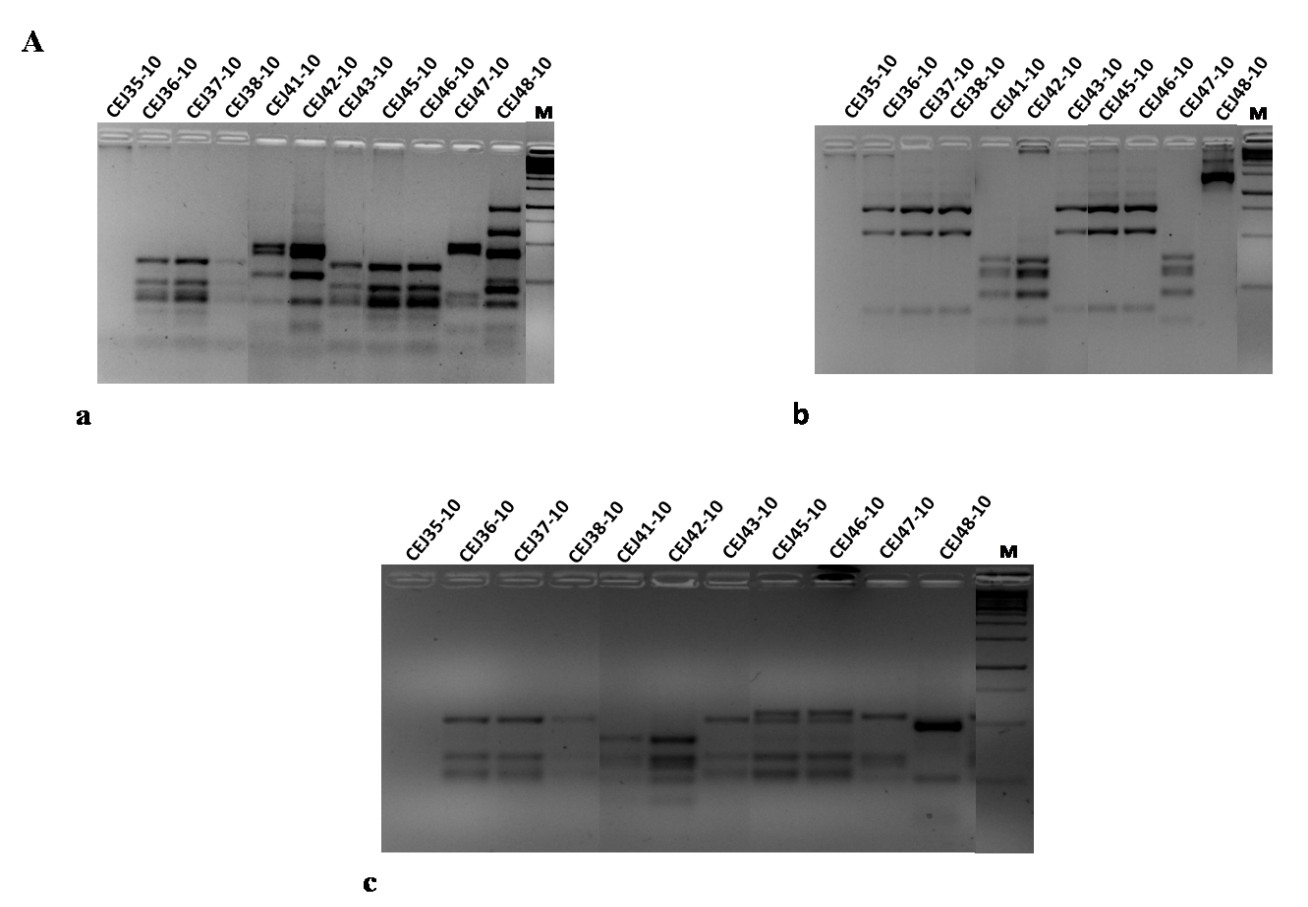


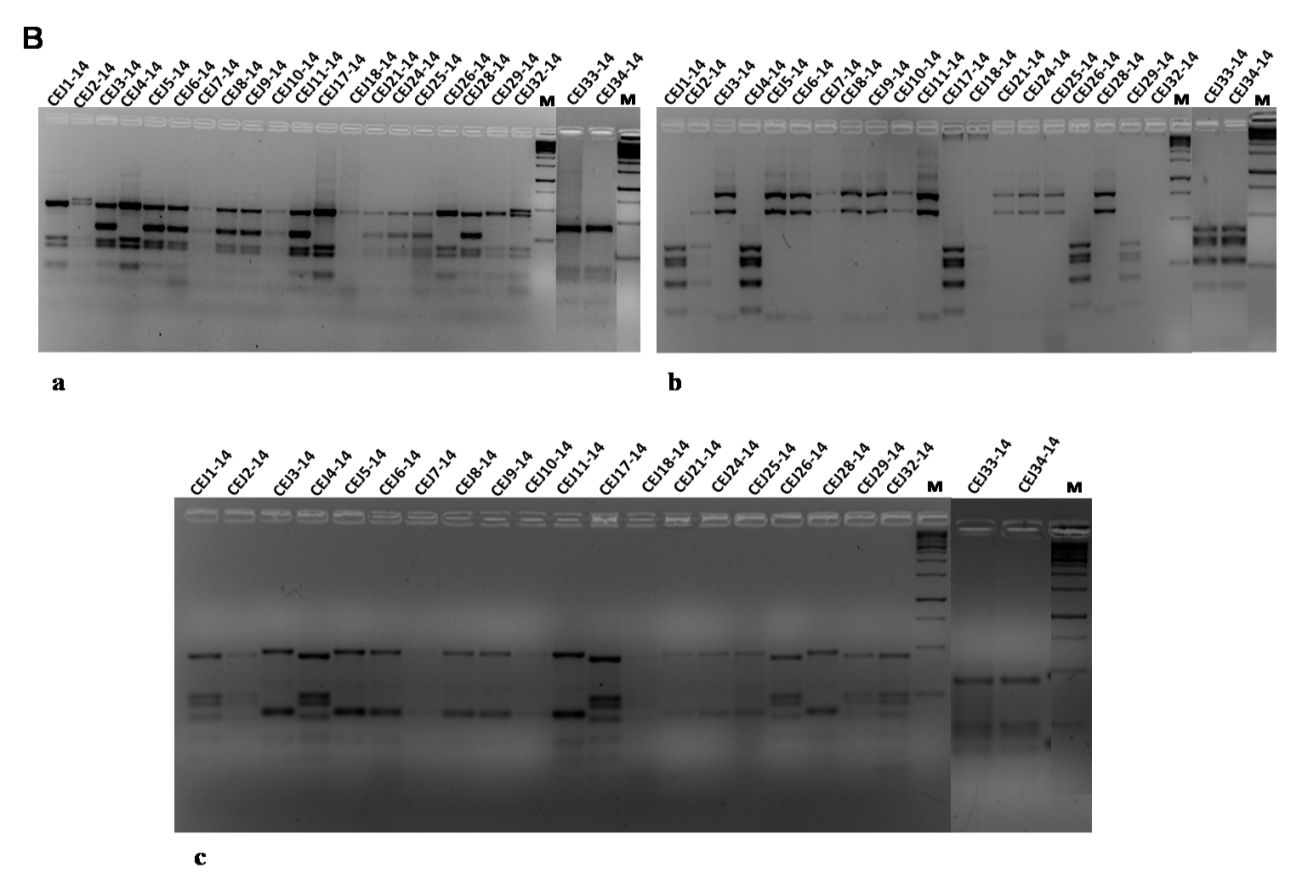


Figure S4: (A) Restriction digestion of amplified 16S rRNA of isolates cleaving with *Alu*I (a), *Mbo*I (b) and *Hae*III (c) from the sample S1-10 and (B) from the samples S6M-14 and S6W-14. Lane M represents molecular size marker 1 Kb DNA ladder.
