# Supplementary material for: Production of Poly(3-Hydroxybutyrate) by Haloarcula, Halorubrum, and Natrinema Haloarchaeal Genera Using Starch as a Carbon Source
Source: Archaea. 2021 Jan 26;2021:8888712. doi: 10.1155/2021/8888712 (PMC7860971; doi:10.1155/2021/8888712)
Supplement: Supplementary 5 — Figure S5: Growth curve over time of the isolates. Optical density was taken every 24 h at 600 nm. Mean values from duplicate tests are shown. [file 8888712.f5.docx]

**
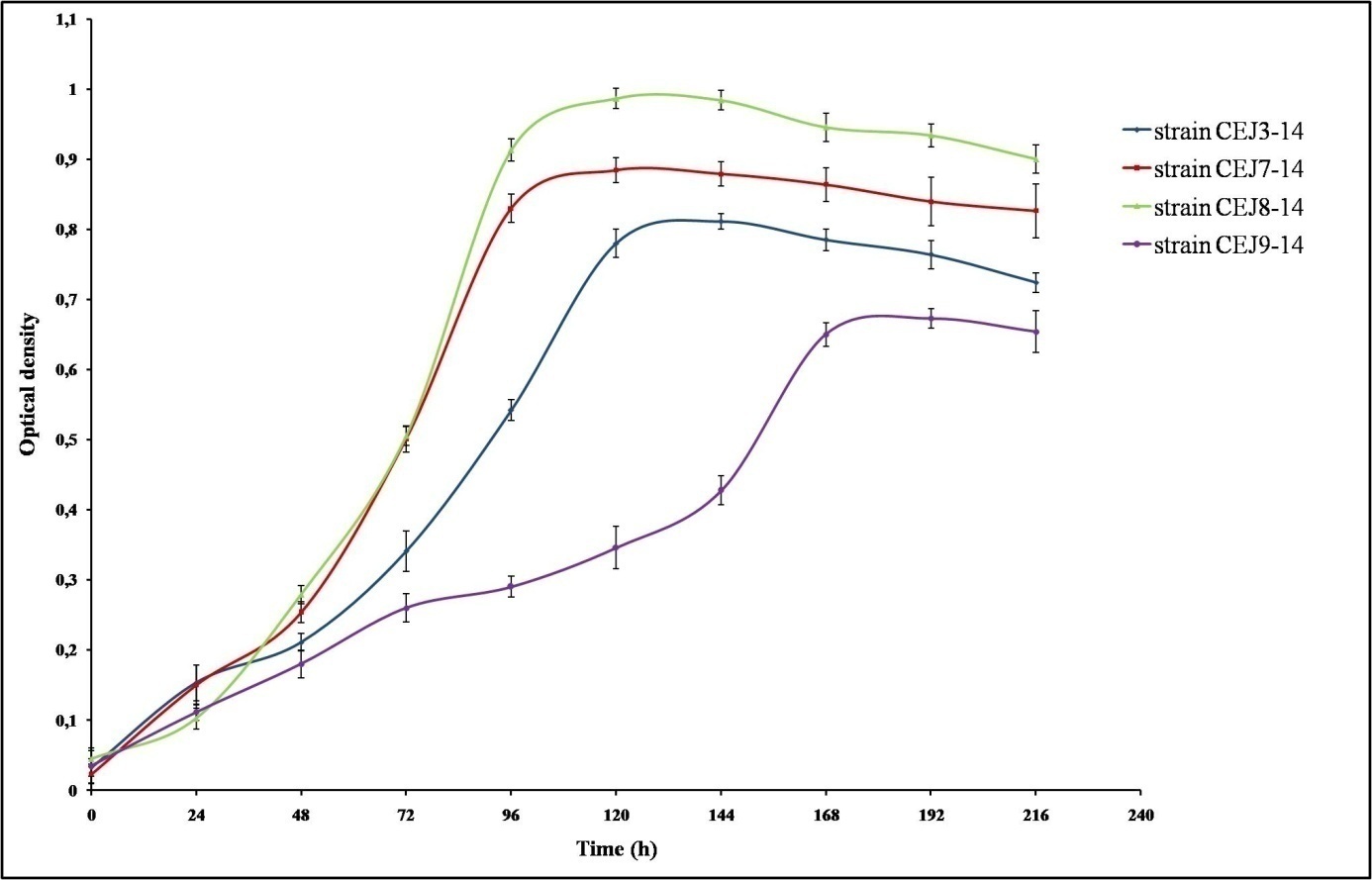
**

**
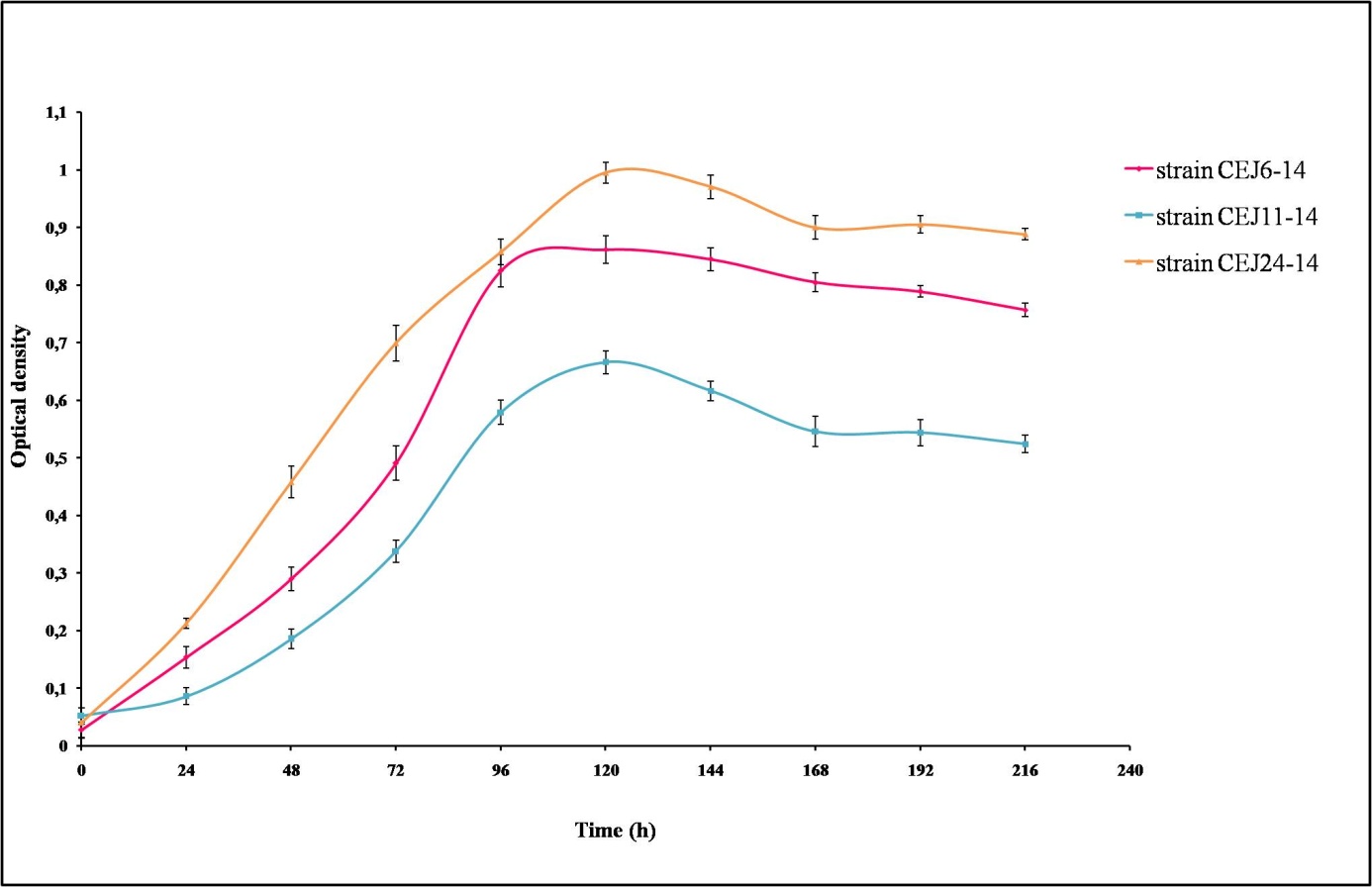
**

**
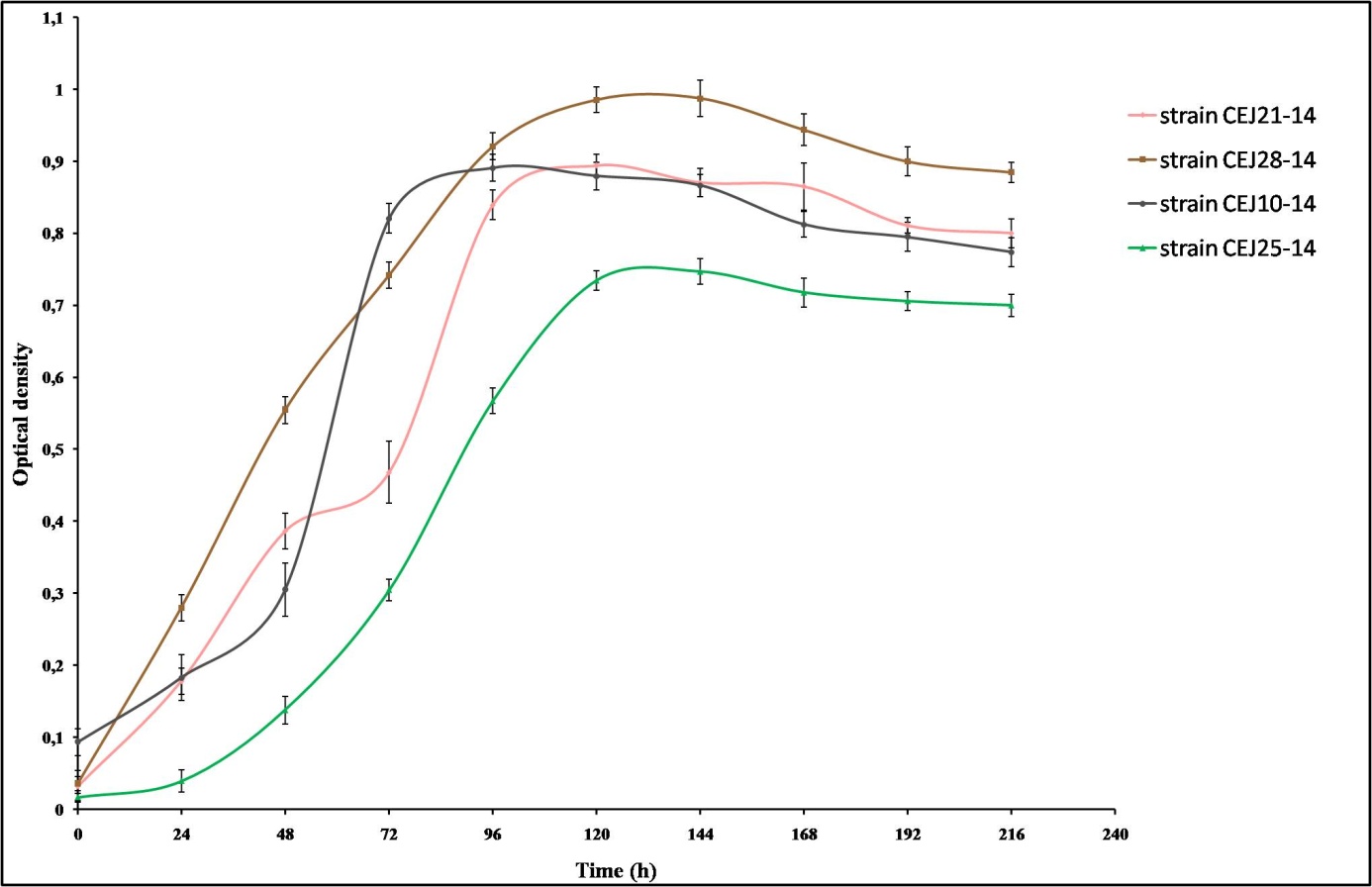
**

**Figure S5:** Growth curve over time of the isolates. Optical density was taken every 24 h at 600 nm. Mean values from duplicate tests are shown.
