# Supplementary material for: Production of Poly(3-Hydroxybutyrate) by Haloarcula, Halorubrum, and Natrinema Haloarchaeal Genera Using Starch as a Carbon Source
Source: Archaea. 2021 Jan 26;2021:8888712. doi: 10.1155/2021/8888712 (PMC7860971; doi:10.1155/2021/8888712)
Supplement: Supplementary 6 — Figure S6: Chromatograms of PHB obtained from cultures of the isolates (a) CEJ3-14, (b) CEJ6-14, (c) CEJ7-14, (d) CEJ8-14, (e) CEJ9-14, (f) CEJ10-14, (g) CEJ11-14, (h) CEJ21-14, (i) CEJ24-14, (j) CEJ25-14, (k) CEJ28-14, and (l) PHB standard (Sigma). [file 8888712.f6.docx]

**
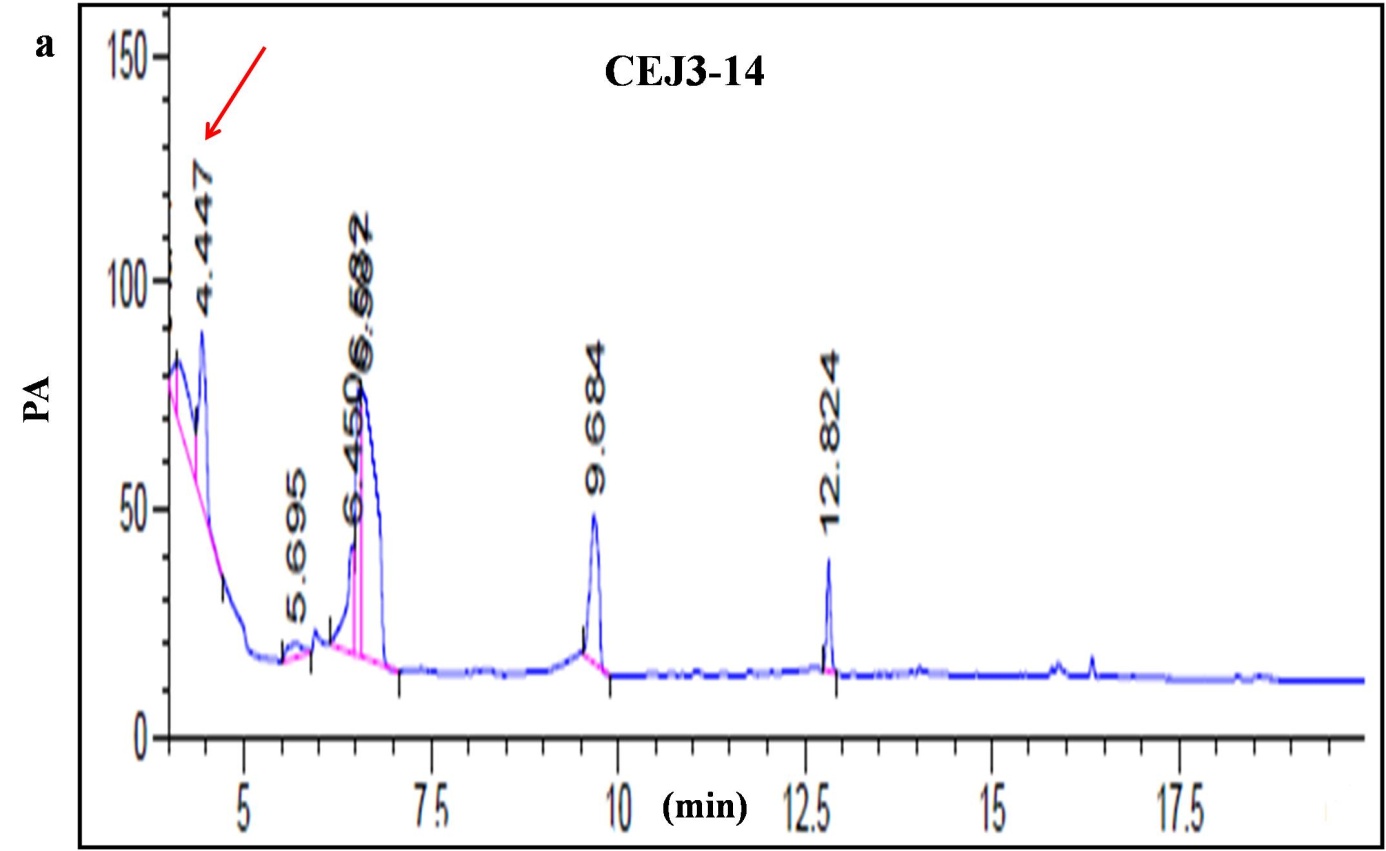
**

**
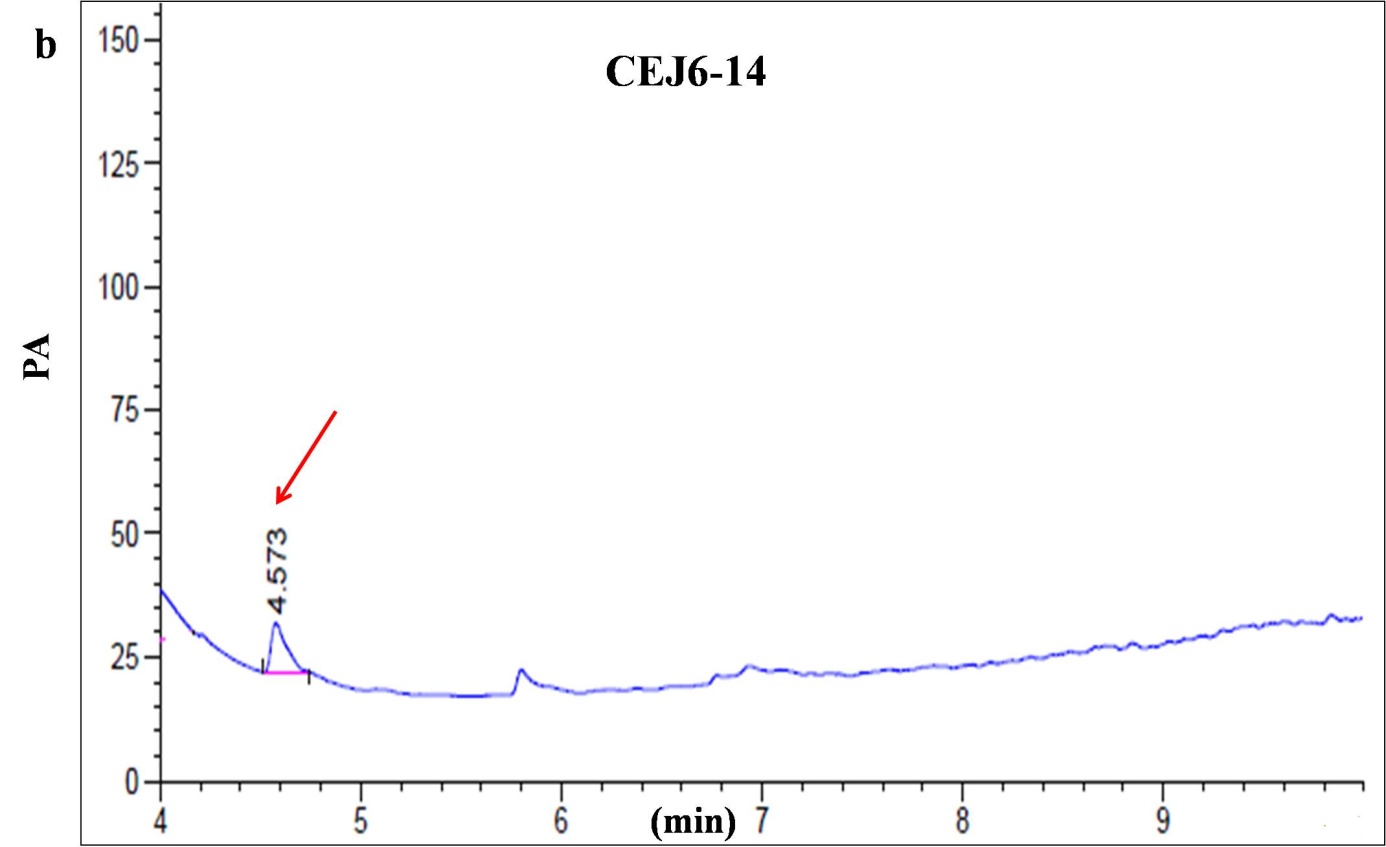
**


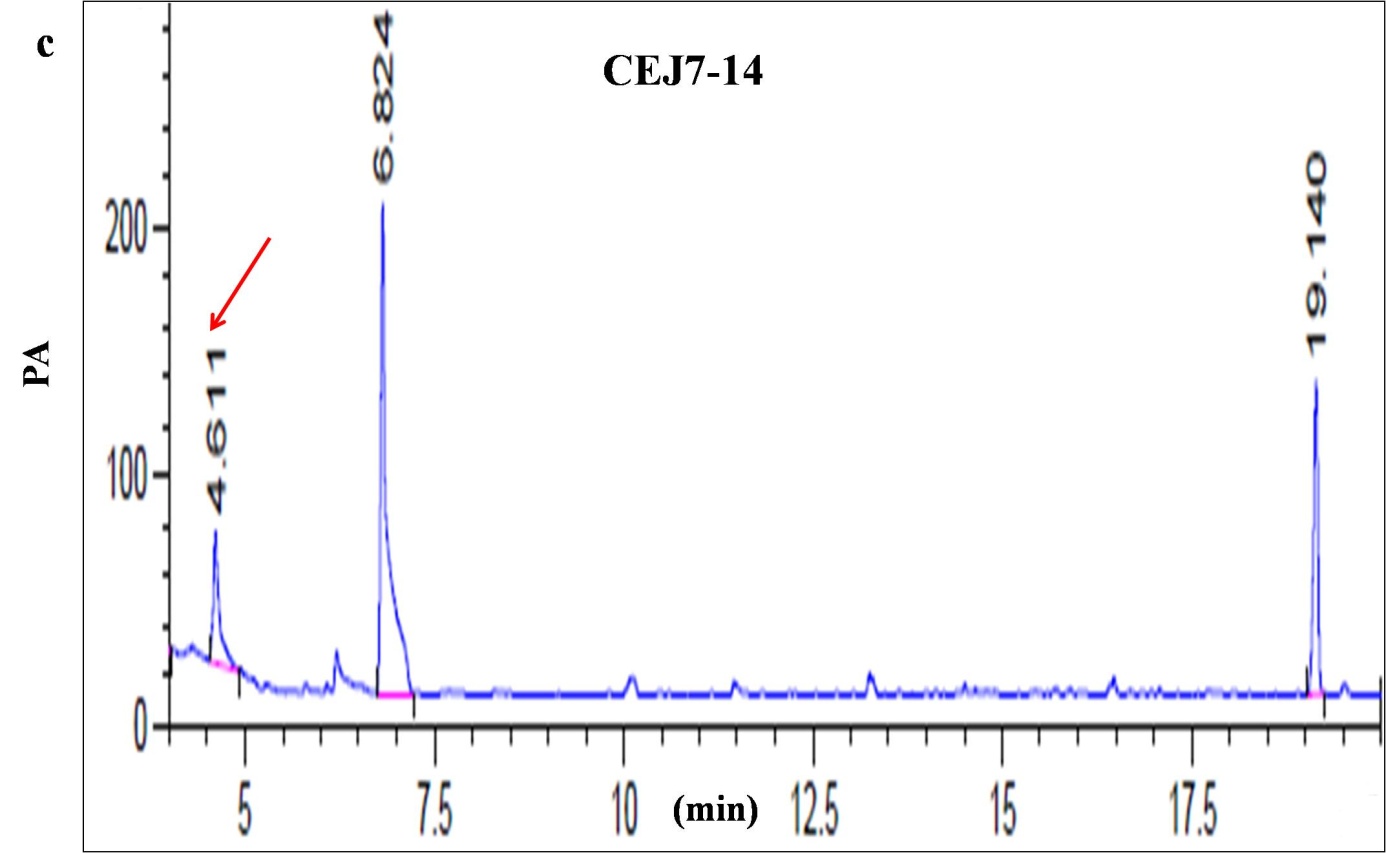


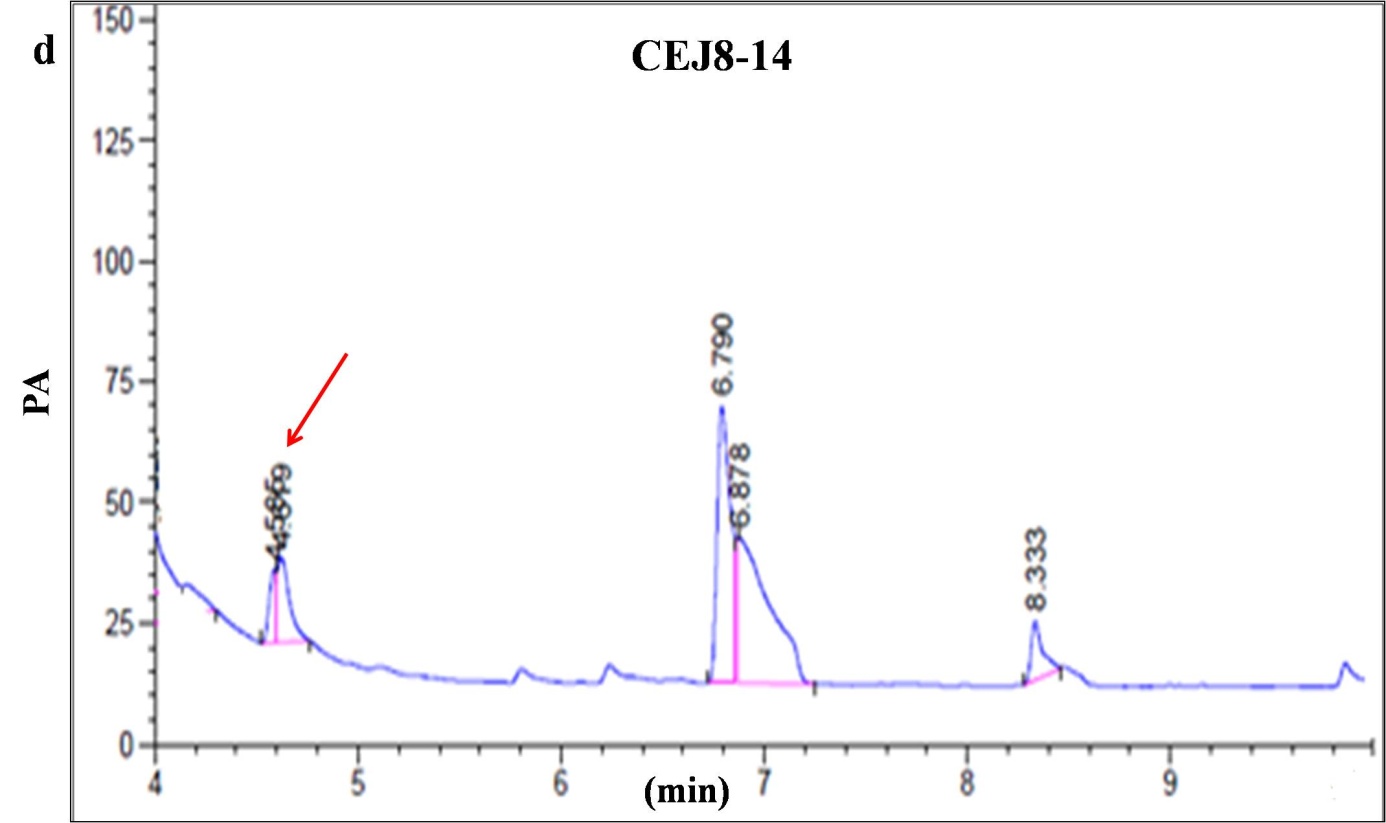


**
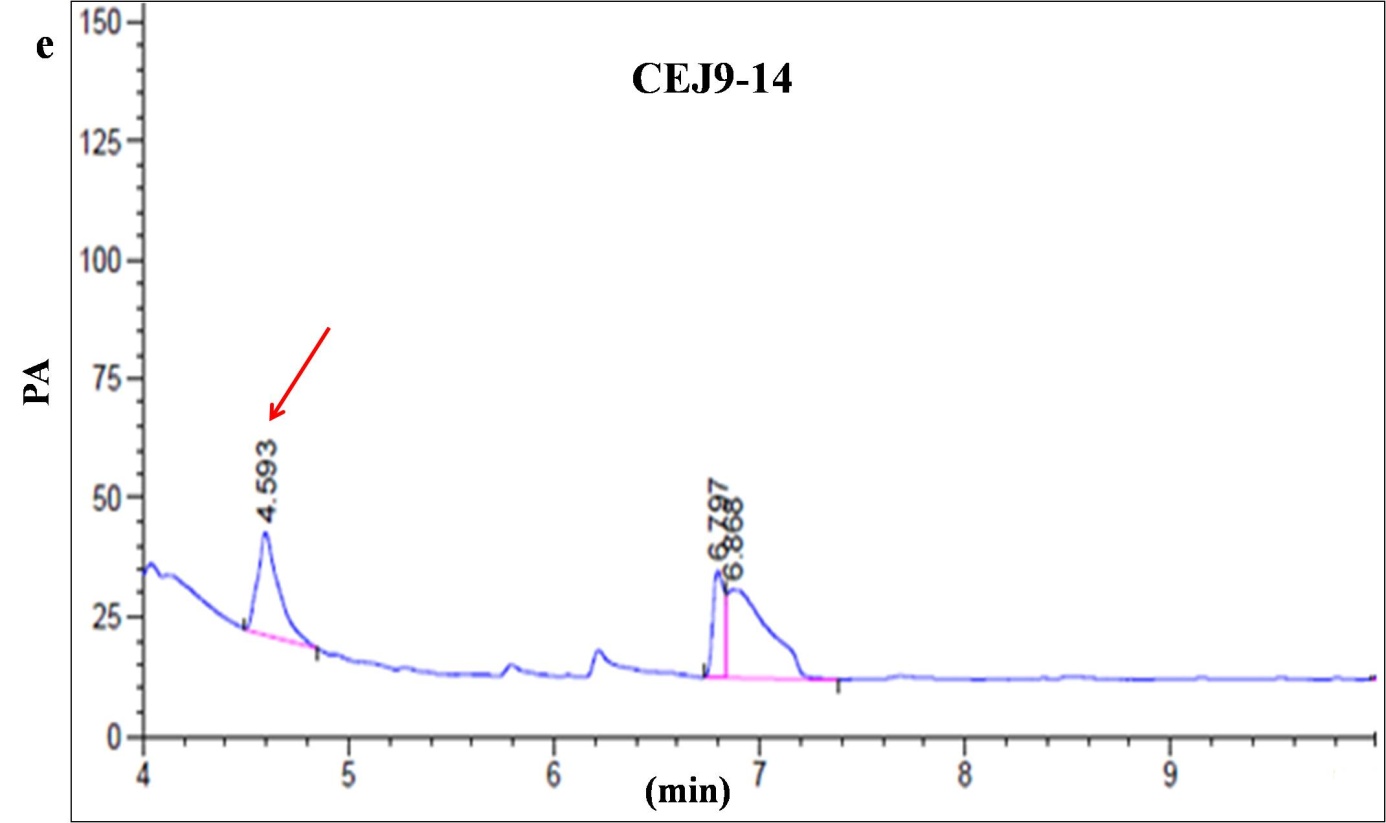
**

**
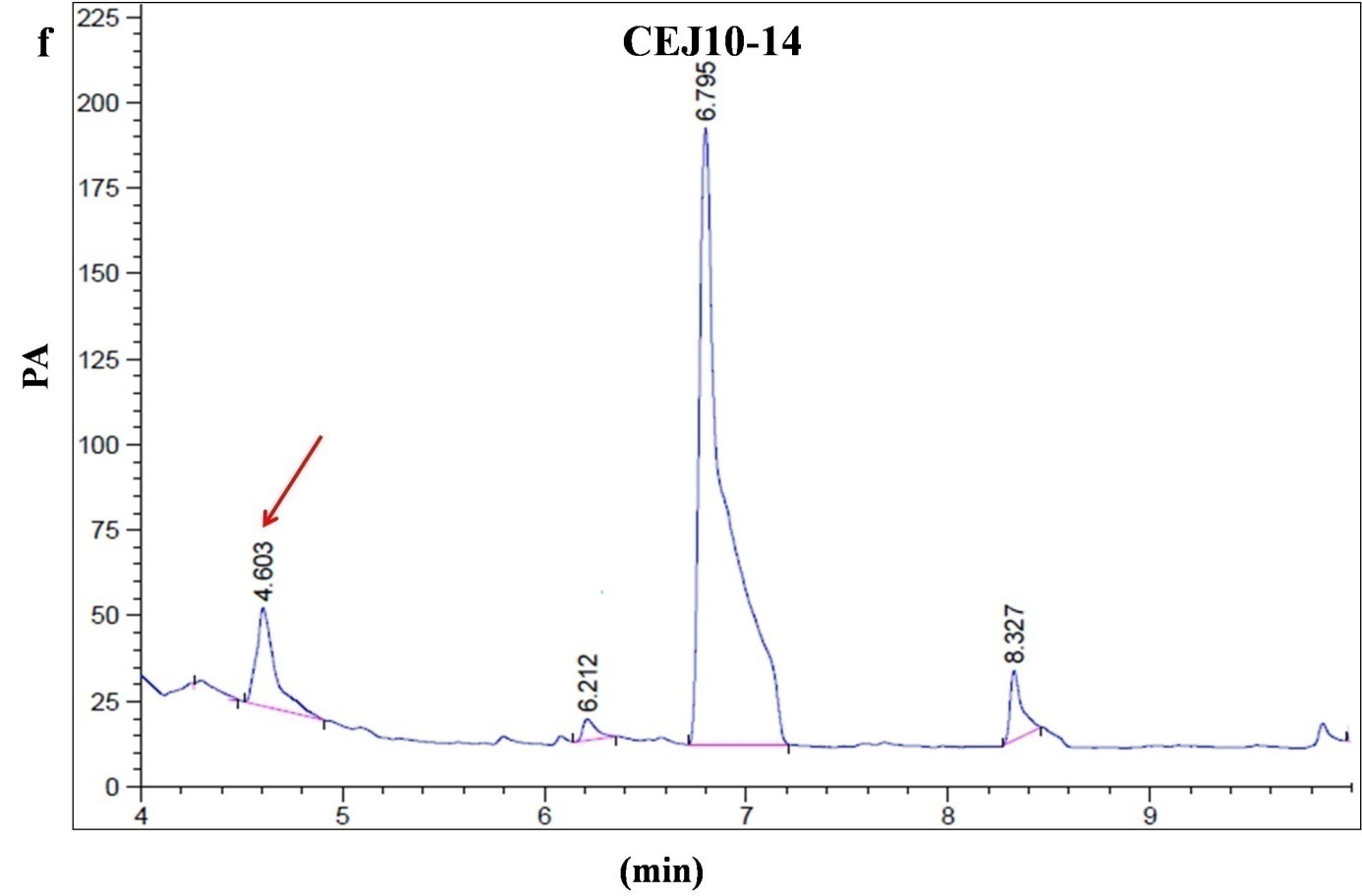
**

**
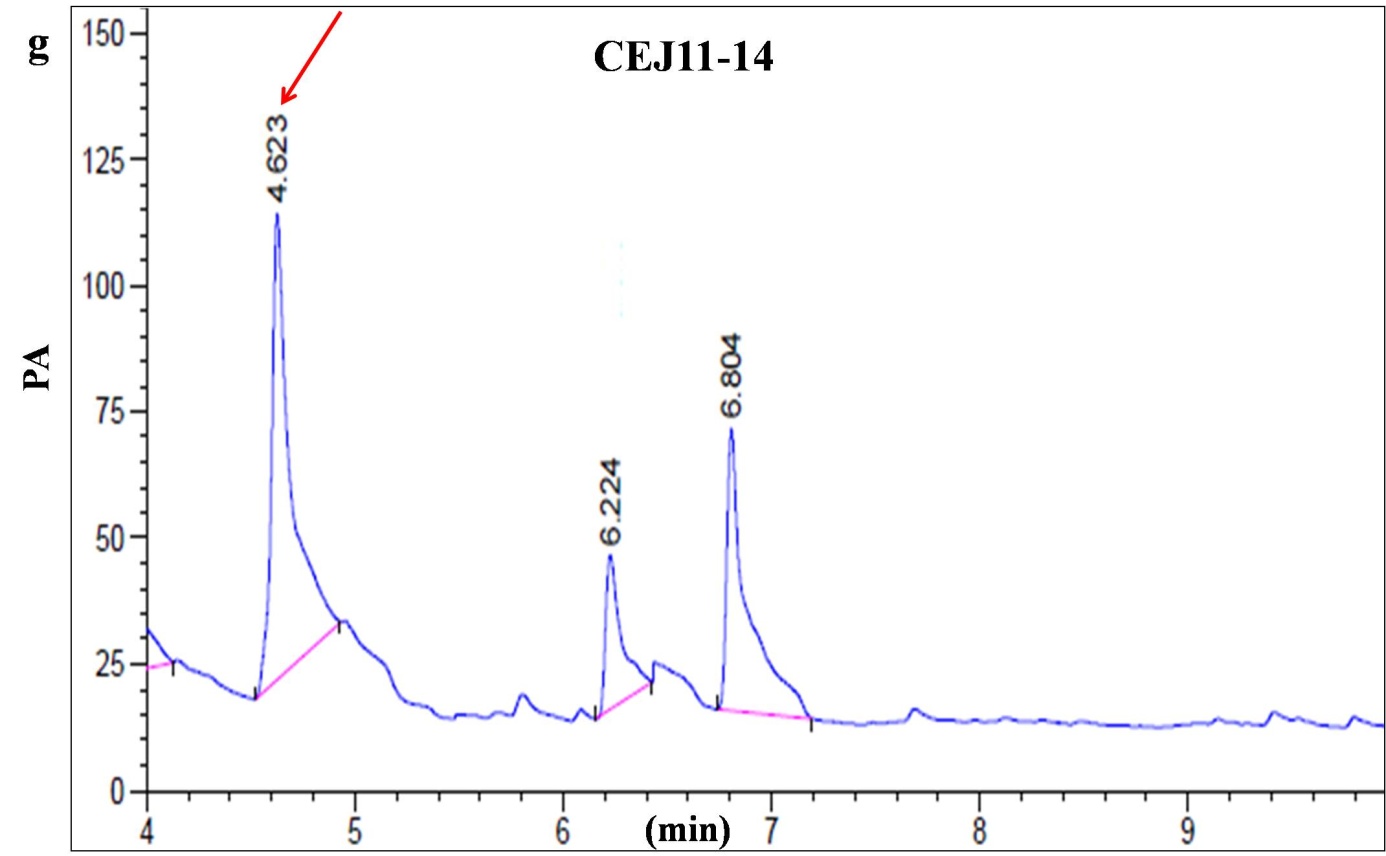
**

**
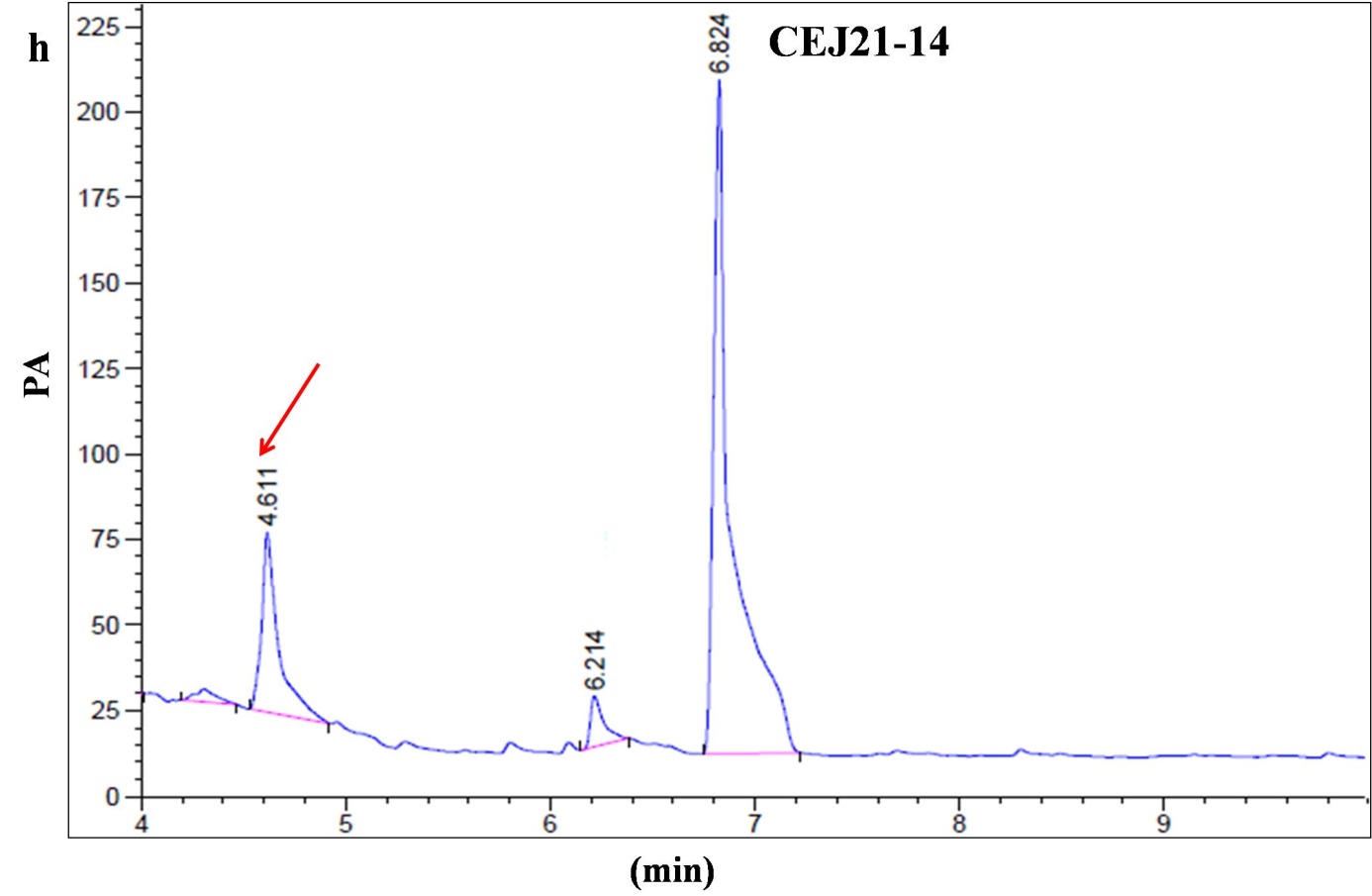
**

**
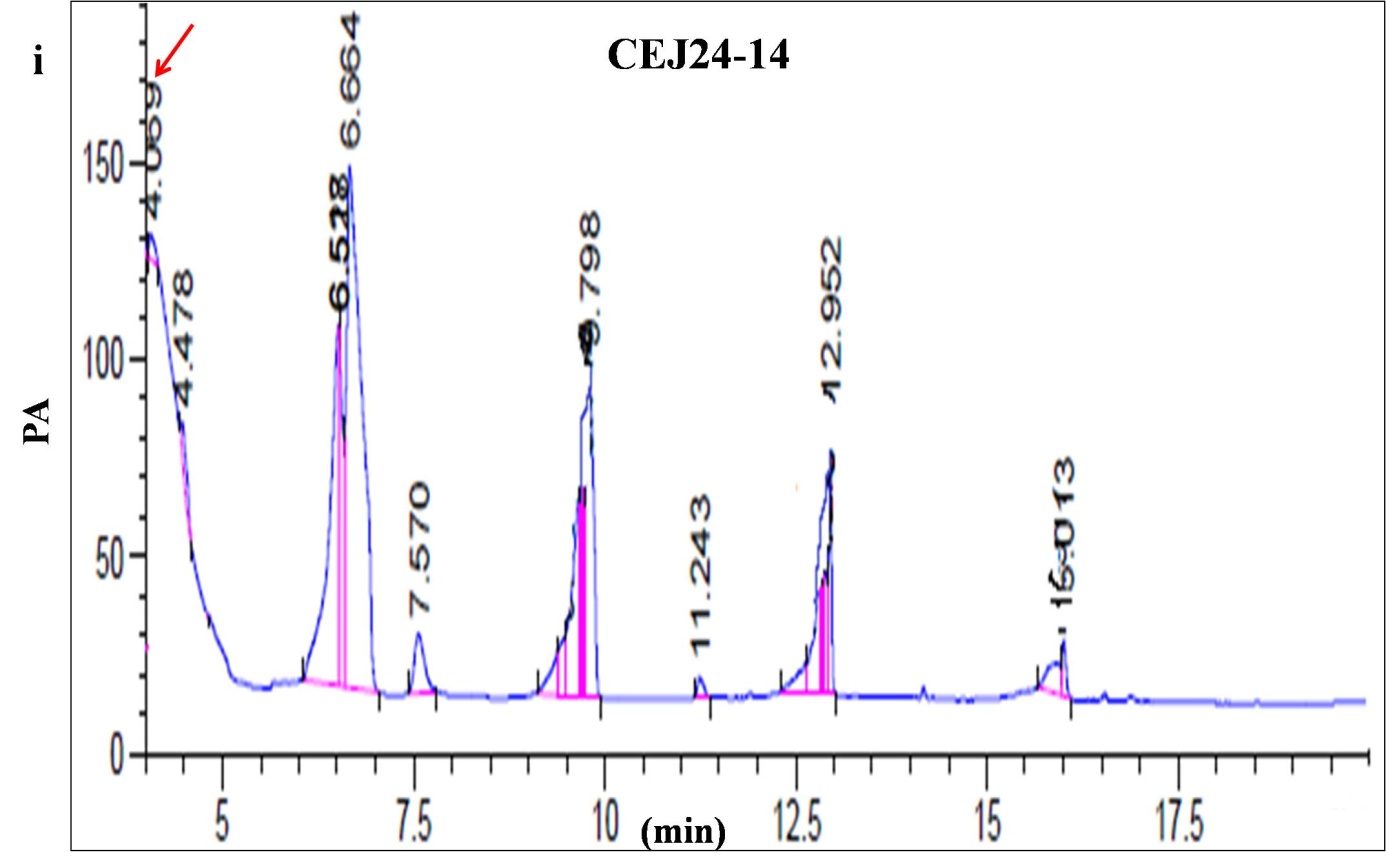
**

**
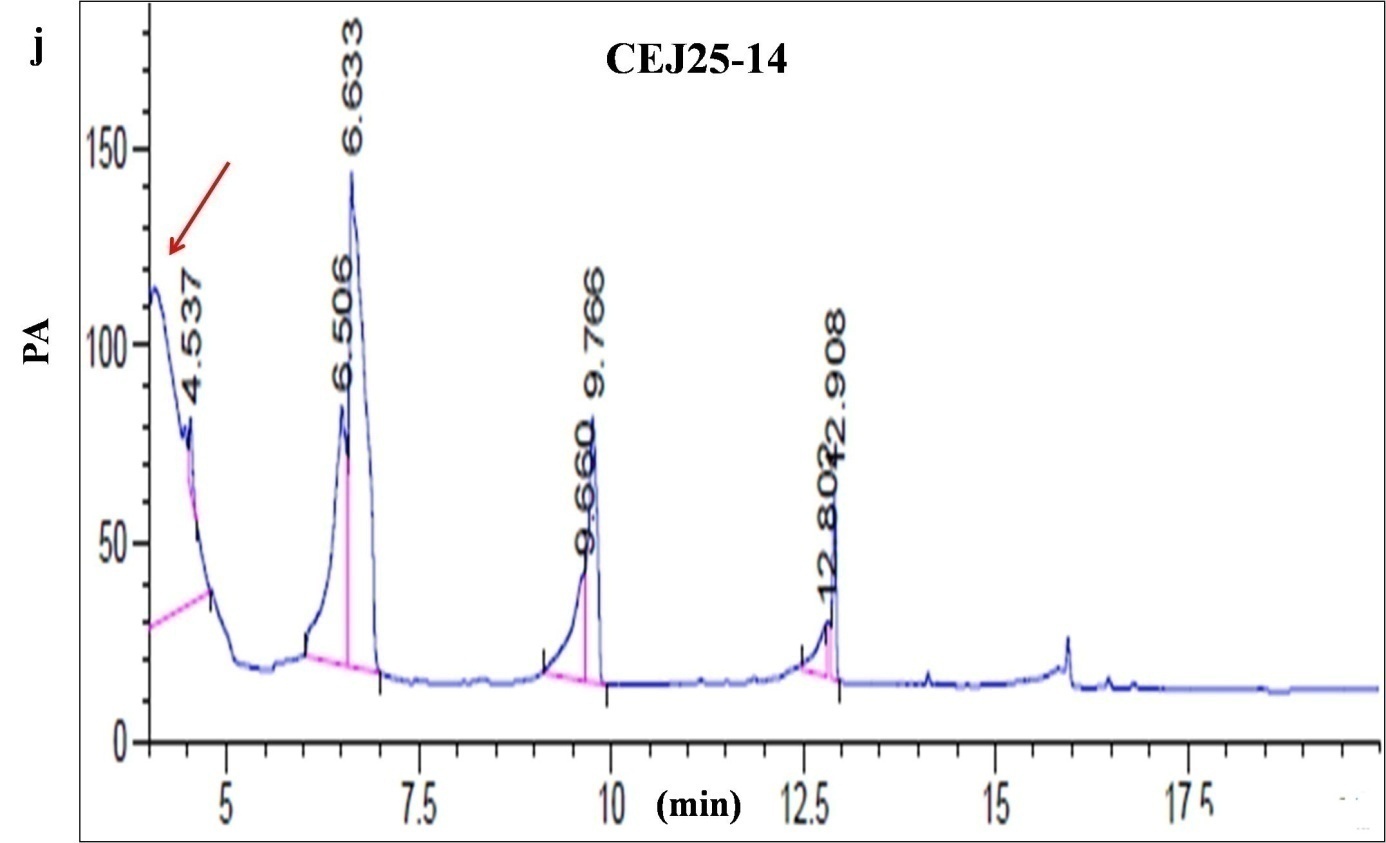
**

**
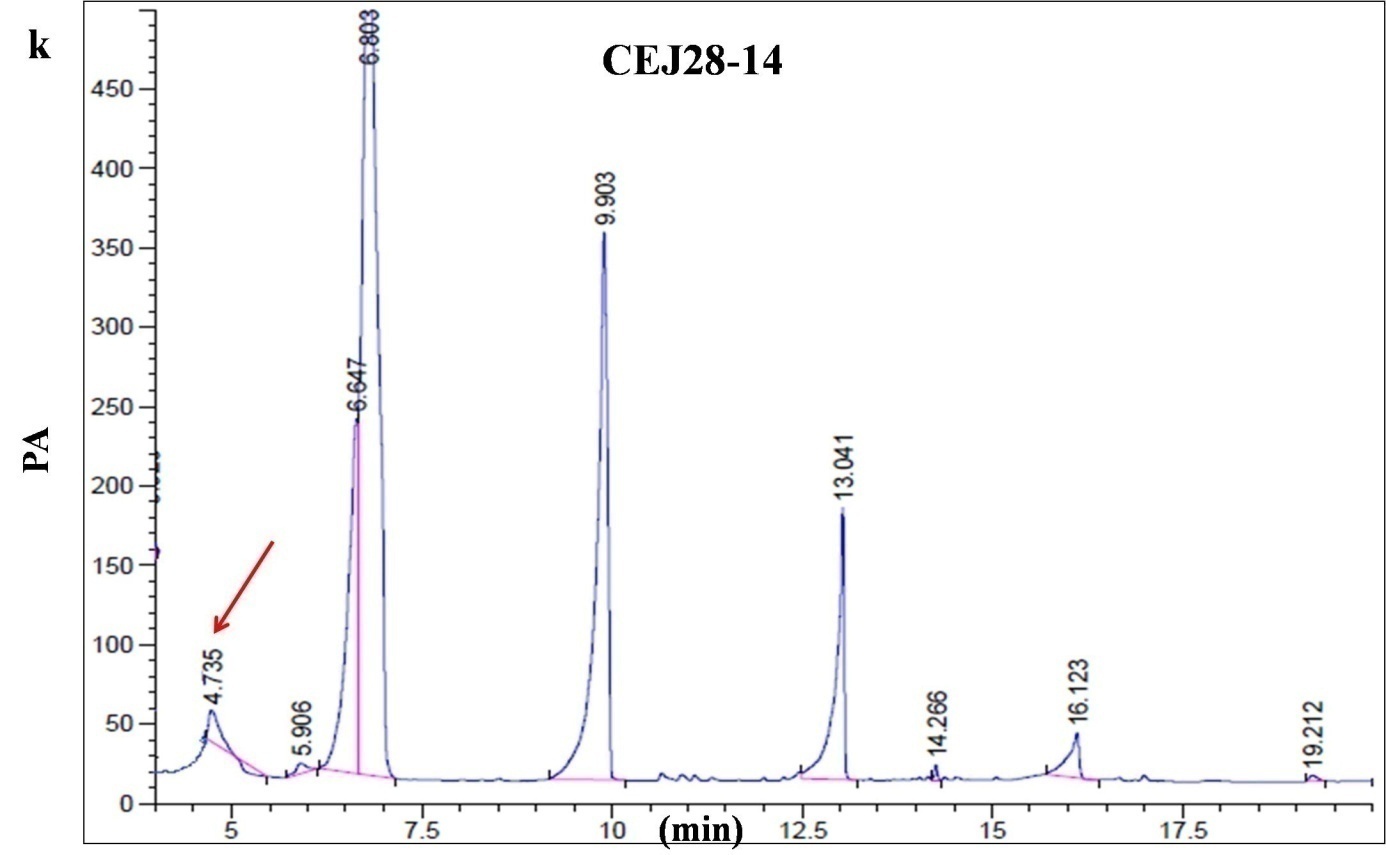
**

**
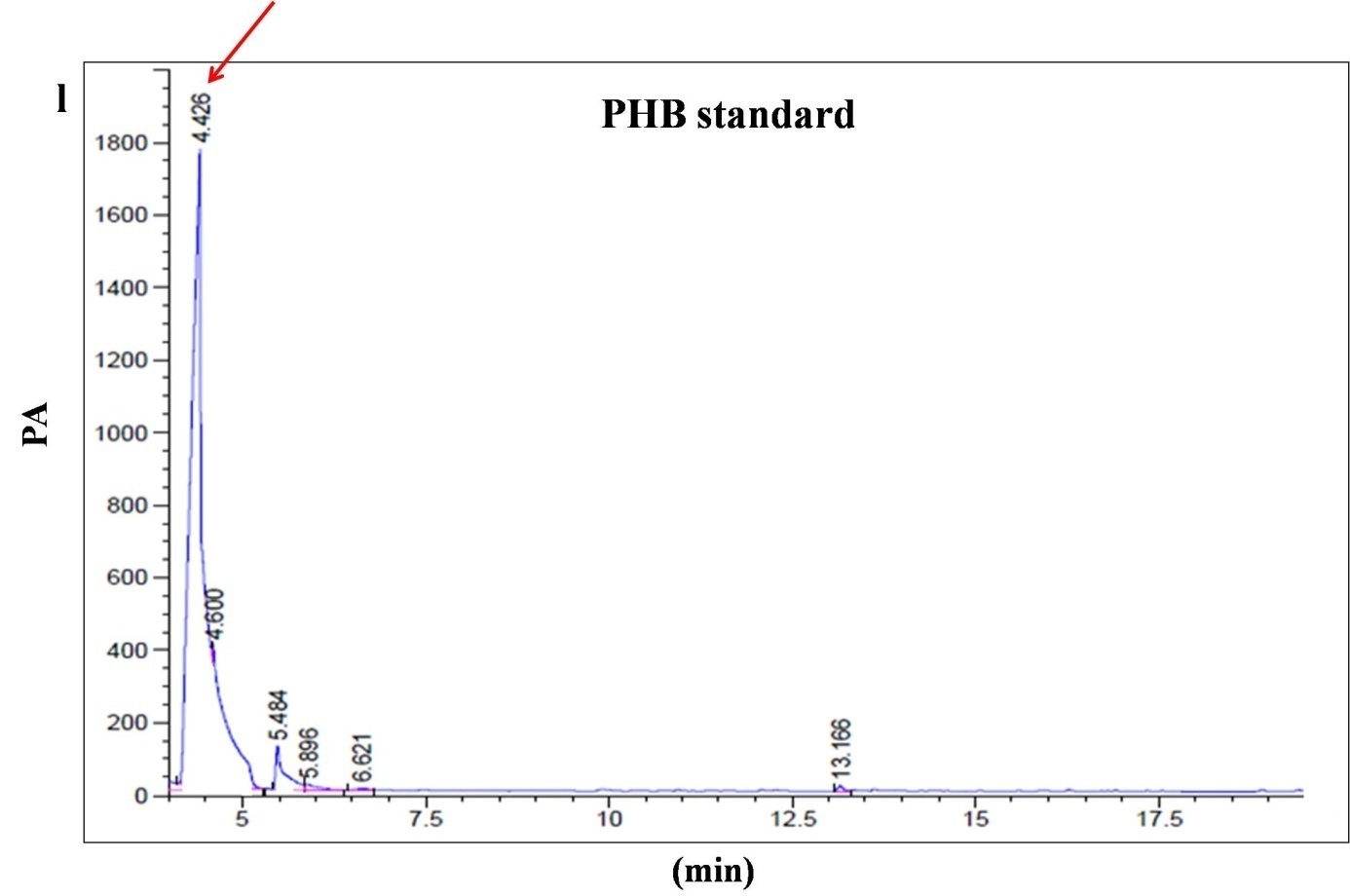
**

**Figure S6:** Chromatograms of PHB obtained from cultures of the isolates (a) CEJ3-14; (b) CEJ6-14; (c) CEJ7-14; (d) CEJ8-14; (e) CEJ9-14; (f) CEJ10-14; (g) CEJ11-14; (h) CEJ21-14; (i) CEJ24-14; (j) CEJ25-14; (k) CEJ28-14; (l) PHB standard (Sigma).
